# Effects of the combination of red yeast rice-containing commercial Chinese polyherbal preparation with statins for dyslipidemia: a systematic review and meta-analysis

**DOI:** 10.3389/fphar.2024.1398934

**Published:** 2024-07-23

**Authors:** Menglong Shi, Tianye Sun, Chenyao Zhang, Yucong Ma, Bo Pang, Lujia Cao, Zhaochen Ji, Fengwen Yang, Junhua Zhang

**Affiliations:** ^1^ Evidence-Based Medicine Center, Tianjin University of Traditional Chinese Medicine, Tianjin, China; ^2^ Dongfang Hospital, Beijing University of Chinese Medicine, Beijing, China; ^3^ Haihe Laboratory of Modern Chinese Medicine, Tianjin University of Traditional Chinese Medicine, Tianjin, China; ^4^ College of Traditional Chinese Medicine, Tianjin University of Traditional Chinese Medicine, Tianjin, China

**Keywords:** red yeast rice, dyslipidaemia, Xuezhikang capsule, Zhibitai capsule, Zhibituo capsule, meta-analysis

## Abstract

**Background:**

Significant challenges are associated with the pharmacological management of dyslipidemia, an important risk factor for cardiovascular disease. Limited reliable evidence exists regarding the efficacy of red yeast rice (RYR)-containing commercial Chinese polyherbal preparation (CCPP), despite their widespread use in China.

**Purpose:**

We aimed to investigate the efficacy of RYR-containing CCPPs combined with statins in treating dyslipidemia.

**Methods:**

Eight databases were searched for relevant randomized controlled trials (RCTs) from database inception date to November 2023. Outcome measures, including low-density lipoprotein cholesterol (LDL-C), high-density lipoprotein cholesterol (HDL-C), total cholesterol (TC), triglyceride (TG), clinical efficacy, and adverse reactions, were assessed. The Cochrane Handbook for Systematic Reviews of Interventions was used for quality evaluation, and the meta-analysis was conducted using RevMan 5.3 and Stata 15.1.

**Results:**

Thirty-three studies involving 4,098 participants were included. The combination of RYR-containing CCPP, such as Xuezhikang (XZK), Zhibitai (ZBTAI), or Zhibituo (ZBTUO) with statins had a significant effect on the increase in clinical efficacy [RR:1.16, 95%CI (1.13, 1.19), *p* < 0.00001]. In addition, they also improved blood lipid profile parameters by increasing HDL-C levels [MD:0.21, 95%CI(0.17, 0.25), *p* < 0.00001], and decreasing TC [MD: 0.60, 95%CI(–0.76, −0.45), *p* < 0.00001], TG [MD: 0.33, 95%CI(–0.39, −0.26), *p* < 0.00001] and LDL-C levels [MD: 0.45, 95%CI(–0.54, −0.36), *p* < 0.00001]. No significant adverse reactions was observed in the RYR-containing CCPPs. Notably, ZBTAI and XZK significantly reduced the incidence of gastrointestinal disturbances and muscular adverse reactions. However, subgroup analyses suggested that the type of CCPPs, dose, and treatment duration might affect the efficacy of RYR-containing CCPPs.

**Conclusion:**

RYR-containing CCPPs combined with statins appears to improve lipid profiles and clinical efficacy in patients with dyslipidemia. However, due to the poor quality of the included studies, and some studied showing negative findings was unpublished. The results should be interpreted with caution until further confirmation by well-designed RCTs.

**Systematic Review Registration:**

https://www.crd.york.ac.uk/prospero/display_record.php?RecordID=487402, identifier CRD42023487402.

## Graphical Abstract

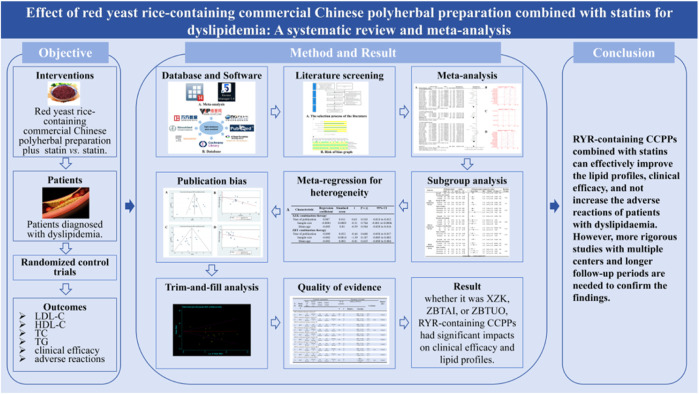

## 1 Introduction

Dyslipidemia, characterized by an abnormal increase in triglyceride (TG), total cholesterol (TC), and low-density lipoprotein cholesterol (LDL-C) levels, and decrease in high-density lipoprotein cholesterol (HDL-C) levels, is a common problem associated with lipid abnormalities (Townsend et al., 2015). Furthermore, dyslipidemia is an important risk factor for cardiovascular disease, which is the leading cause of morbidity and mortality worldwide (Visseren et al., 2021). Altered lipid profiles have significantly contributed to improving cardiovascular disease (CVD), with a survey in United States indicating that the death rate associated with coronary heart disease decreased by more than 40 percent from 1980 to 2000, with a reduction in TC levels being the largest contributor. Therefore, the control of dyslipidemia is the key to prevent CVDs (Ford et al., 2007). However, epidemiological studies have found that the global prevalence of dyslipidemia was 15.2% in 2019 (Zeljkovic et al., 2019). In addition, the prevalence of dyslipidemia among adults in China was 40.45% in 2012, representing a significant increase over the previous period (World Health Organization, 2020). Even among those identified with arteriosclerotic cardiovascular disease (ASCVD) or at high risk of ASCVD, only 26.6% and 42.9%, respectively, exhibited LDL-C control targets (Dai et al., 2022). Furthermore, dyslipidemia has been regarded as a major causative factor for many diseases, such as cerebral infarction, hypertension, and kidney dysfunction. In summary, it is important to manage and control blood lipid levels.

Currently, dyslipidemia management involves drug management and lifestyle changes (Gerhard-Herman, 2017; Whelton et al., 2018). Commonly prescribed medications for dyslipidemia include statins, cholesterol absorption inhibitors, absorption inhibitors, cholic acid chelating agents, fibrates, and nicotinic acid. Among these, statins are the basic drugs for the treatment of dyslipidemia (Mach et al., 2020). Although drug management has achieved positive results in lowering lipid levels, it remains a challenge due to adverse reactions such as neuropathy, gastrointestinal reaction, and related muscle complications such as myalgia, myositis, myopathy, or rhabdomyolysis (Stroes et al., 2015). Moreover, research showed that the use of statins, especially at large doses or for long durations, is related to an increased risk of myopathy, new-onset diabetes mellitus, and, probably, haemorrhagic stroke (Collins et al., 2016; Pergolizzi et al., 2020). Notably, unsatisfactory therapeutic effects and patient compliance-related issues also affect lipid management (Soppert et al., 2020). Therefore, it is necessary to explore additional therapies to achieve improved dyslipidemia treatment.

The available evidence suggests that traditional Chinese medicine, especially red yeast rice (RYR), in the treatment of dyslipidemia is increasingly recognized (Hu et al., 2022). RYR is a type of fermented rice produced by the fermentation of Monascus purpureus, and its active metabolites, including monacolin, can effectively regulate lipid levels (Jiang et al., 2021; Banach et al., 2022; Buzzelli et al., 2023). The Zhibitai capsule (ZBTAI), Zhibituo capsule (ZBTUO), and Xuezhikang capsule (XZK), which are RYR-containing CCPPs, are orally administered drugs approved by the Chinese State Food and Drug Administration (Management, 2023). Studies have confirmed that RYR-containing CCPPs can effectively reduce blood lipid levels in patients. Additionally, they can reduced the mortality rate in patients with coronary heart disease (Cicero et al., 2023). Notably, XZK is described as a medium-intensity lipid-lowering drug with proven safety that can significantly reduce LDL-C (Lu et al., 2008; Mach et al., 2020). Additionally, the results of multi-center clinical trials have demonstrated that ZBTAI combined with statins was as effective in reducing LDL-C levels as high-dose statins alone (Xu et al., 2018). The botanical drugs included and traditional effects of RYR-containing CCPPs were described in Supplementary Table S2.

The widespread use of RYR-containing CCPPs has resulted in an increase in the number of systematic reviews (SRs) to assess their efficacy (Wang et al., 2022; Zhao et al., 2023). However, these SRs was found several shortcomings such as heterogeneity and publication bias, which have not been addressed and explained. It is worth noting that there were significant differences in the interventions of the control group included in the previous systematic review, which reduced the statistical reliability. Therefore, statins, currently the basic drug for the treatment of dyslipidemia, were selected as the control group in this study, aiming to provide a new evaluation of the efficacy of RYR-containing CCPPs combined with statins in the treatment of dyslipidemia.

## 2 Methods

This study strictly followed the Preferred Reporting Program for Systematic Review and Meta-Analysis (PRISMA) guidelines (Page et al., 2018) and has been registered in the International Prospective Register of Systematic Reviews (registration No. CRD42023487402).

### 2.1 Search strategy

A comprehensive search for all relevant studies was performed in China Biomedical Literature Service System, the Chinese Science and Technology Journals Database (VIP), China National Knowledge Infrastructure (CNKI), Wanfang Data, PubMed, Embase, CENTRAL, and Web of Science from database inception date to 22 November 2023 by two researchers (STY and ZCY). No language or geographical areas restrictions were put in place. Relevant keywords containing both medical subject headings and free text terms. Keywords for the intervention included “Red yeast rice”, “Xurzhikang”, “Zhibitai”, “Zhibituo”, and “Monascus”, while keywords for the study population were “Dyslipidemias”, “Hyperlipidemias”, “Hypercholesterolemia”, “Hyperlipoproteinemia”, and “Hyperlipidemia, Familial Combined”. Furthermore, potential missing studies were further identified by reviewing references of these studies. (The detailed search strategies are shown in Supplementary Table S3).

### 2.2 Inclusion criteria

#### 2.2.1 Types of patients

Patients with dyslipidemia meeting accepted diagnostic criteria were included (Management, 2023), without age, sex, race, complications, or type of dyslipidemia restriction. Diagnostic criteria include either TC ≥ 6.2 mmol/L or TG of  ≥ 2.3 mmol/dL, or LDL-C   ≥   4.1 mmol/L, or HDL-C  < 1.0 mmol/L.

#### 2.2.2 Intervention and control

The control group was treated with statins alone (fluvastatin, lovastatin, pitavastatin, rosuvastatin, and simvastatin). Participants in the experimental group were administered a combination of RYR-containing CCPPs and statins. The doses and statin types in the control and experimental groups were the same.

#### 2.2.3 Outcome measures

Primary outcomes included low-density lipoprotein cholesterol (LDL-C), high-density lipoprotein cholesterol (HDL-C), total cholesterol (TC), and triglyceride (TG) levels. Additional valuable outcomes that can help obtain accurate data were also collected. These included (1) clinical efficacy; (2) other lipid profiles, such as apolipoprotein A1 (ApoA1) levels, apolipoprotein B (ApoB) levels, and (3) adverse reactions such as muscular adverse drug reactions, kidney dysfunction, and gastrointestinal reactions.

#### 2.2.4 Types of studies

This meta analysis included randomized controlled trials (RCTs) that compared the combination of RYR-containing CCPPs and statins against statins alone.

### 2.3 Exclusion criteria

(1) Non-RCTs, conferences abstracts, animal researches, and technical results; (2) studies with incomplete or inadequate data; (3) interventions involving other Chinese medicines, or therapies specific to Chinese medicine.

### 2.4 Literature screening and data extraction

Based on the inclusion and exclusion criteria, two authors (SML and PB) independently screened studies and extracted the following information: (1) sample characteristics and study design, including authors, publication year, dyslipidemia type, CCPP type, statin type, CCPP dose, and statin dose; (2) outcome information, encompassing the lipid profiles, adverse reactions, and other valuable outcomes. Disagreements were examined by a third researcher (MYC). Attempts were made to contact authors to obtain missing data.

### 2.5 Quality assessment

The Cochrane risk of bias tool 2.0, which includes the randomization sequence generation, deviations from intended interventions, missing outcome data, outcome measurement, and overall bias, was used to assess the quality of included studies. Two authors evaluated each domain independently, and the results were assessed by a third researcher if they were inconsistent.

### 2.6 Data synthesis

Meta-analysis was completed by RevMan 5.3 and Stata 15.1. Relative risk (RR) and mean difference [MD) were determined to evaluate dichotomous data and continuous variables, respectively. Confidence intervals were (CIs) set at 95% and the statistical significance was set at *p* < 0.05. Statistical heterogeneity was conducted by Q test and inconsistency index (*I* ^*2*^) values. If the heterogeneity was obvious (50% < *I* ^*2*^ and 0.1 > *P*), a random-effects model was used, otherwise using the fixed-effects model. Funnel plots and Egger’s test were used to assess the publication bias when the number of included studies exceeded 10. Simultaneously, the influence of publication bias on the results interpretation was evaluated by trim-and-fill analysis. Pre-defined subgroup analysis was first performed to assess the influence of CCPP type on the efficacy of RYR-containing CCPPs. Then, subgroup analyses were performed for different CCPP types to evaluate the influence of some parameters (CCPP dose, statin type, and duration of treatment) on the efficacy of different RYR-containing CCPPs, respectively. Sensitivity analysis was conducted by item-by-item elimination to assess robustness of the meta-analysis. Furthermore, univariate meta-regression analyses were performed to investigate the source of heterogeneity.\

### 2.7 Quality of evidence and evaluation of this SR

Grading of recommendation, assessment, development, and evaluation (GRADE) guidelines to assess the certainty of the evidence for each outcome, in which five domains were evaluated: (1) study limitations were assessed according to RoB2.0; (2) consistency was evaluated using I^2^ values and the agreement of 95% confidence and prediction intervals; (3) directness was assessed to determine whether the interventions and populations of the included studies were appropriate for the research question; (4) precision was examined by the optimal information sample size; and (5) publication bias was assessed using the funnel plot and the number of included studies (Gonzalez-Padilla and Dahm, 2021). Furthermore, the Modified Quality Assessment Scale for Systematic Reviews (AMSTAR-2) (Shea et al., 2017) and Risk of Bias in Systematic Reviews (ROBIS) tool (Whiting et al., 2016) were used to evaluate the methodological quality and risk of bias of meta-analysis by two investigators (ZP and MYY) who had no conflict of interest with this study. More importantly, meta-analysis was refined according to the review results until each domain are satisfactory.

## 3 Results

### 3.1 Study selection

**FIGURE 1:**
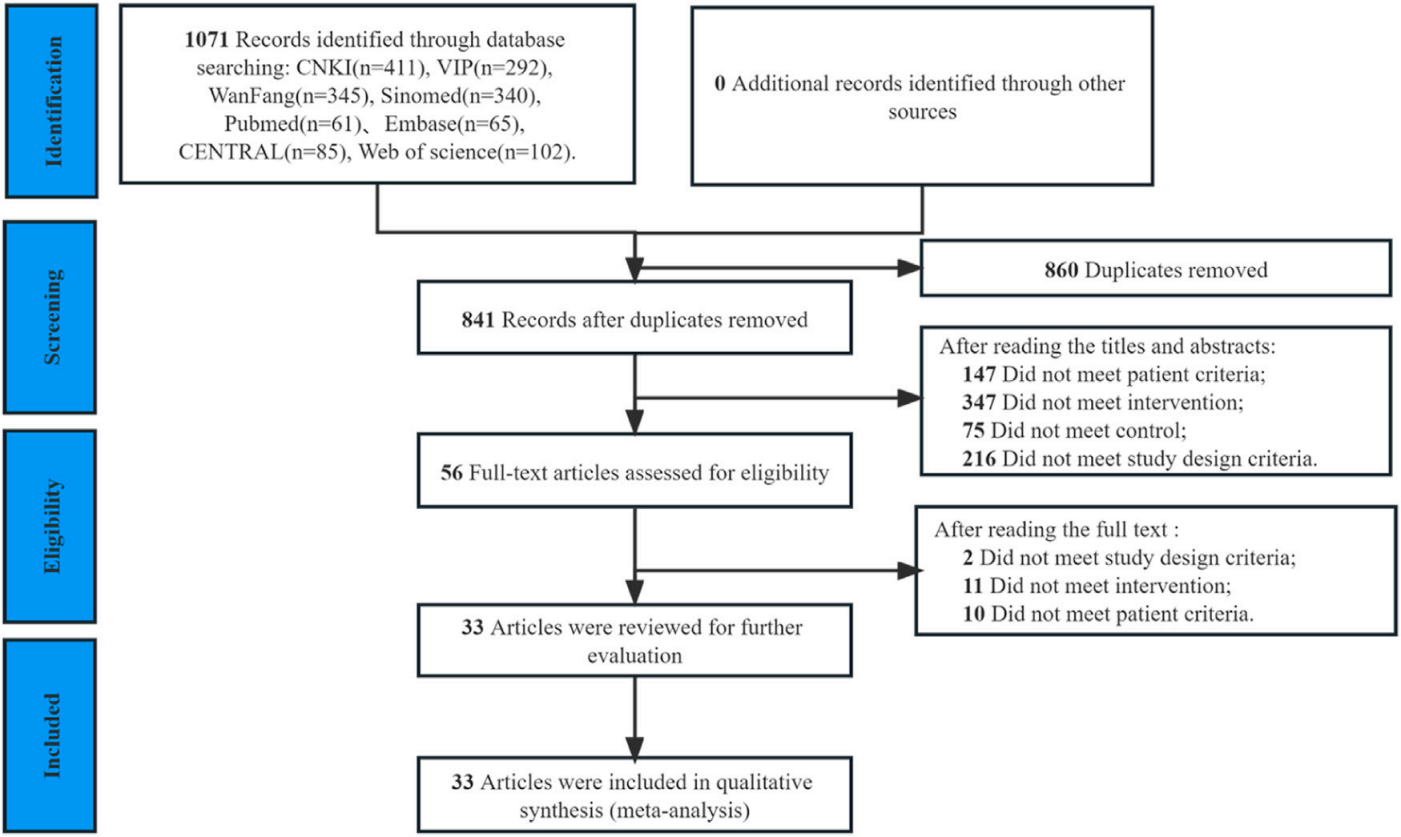
PRISMA flow diagram of study selection.

Out of the 1,735 articles initially identified in the database search, 860 duplicates were removed, and an additional 785 articles were excluded after reviewing titles or abstracts. The full text of 56 trials were reviewed, and 23 were excluded (Supplementary Table S5). Ultimately, 33 RCTs including 4,098 patients (2,048 patients in experimental groups and 2,050 in control groups) (Zhang and Tang, 2010; Zhou, 2010; Ji and Yi, 2011; Jiang and Chen, 2012; Wang, 2012; Zhang et al., 2013; Feng, 2015; Wang and Chen, 2015; Chen et al., 2016; Li et al., 2016; Whiting et al., 2016; Fu, 2017; Zhang et al., 2017; Zou, 2017; Liu et al., 2018; Ma and Feng, 2018; Shi et al., 2018; Sun et al., 2018; Liu, 2019; Liu et al., 2019; Ma and Deng, 2019; Shi, 2019; Xiong, 2019; Chen et al., 2020; He, 2020; Qu et al., 2020; Tan, 2020; Su, 2021; Tan et al., 2021; Yu, 2021; Chen et al., 2022; Tian et al., 2022; Li, 2023; Yuan et al., 2023) were included in the final review (Figure 1).

### 3.2 Studies characteristics

The characteristics of the included studies are indicated in Table 1. All trials were conducted in China and published in Chinese between 2010 and 2023. In terms of disease subtypes, one trial recruited only patients with hypertriglyceridemia (Ma and Deng, 2019), while the remaining studies did not specify the dyslipidemia type. Patients receiving combination therapy with RYR-containing CCPPs categorized into the experimental group and those receiving statin therapy categorized into the control group. In the experimental groups, fourteen trials (Zhang and Tang, 2010; Zhou, 2010; Jiang and Chen, 2012; Wang, 2012; Fu, 2017; Zhang et al., 2017; Zou, 2017; Liu et al., 2018; Shi et al., 2018; Sun et al., 2018; Ma and Deng, 2019; Qu et al., 2020; Yu, 2021; Tian et al., 2022) with 2,094 participants focused on the XZK combination therapy, while fifteen trials ( Wang and Chen, 2015; Chen et al., 2016; Li et al., 2016; Ma and Feng, 2018; Liu, 2019; Liu et al., 2019; Shi, 2019; Xiong, 2019; Chen et al., 2020; He, 2020; Tan, 2020; Tan et al., 2021; Chen et al., 2022; Li, 2023; Yuan et al., 2023) with 1,482 participants focused on the ZBTAI combination therapy, and four trials (Ji and Yi, 2011; Zhang et al., 2013; Feng, 2015; Su, 2021) with 522 participants focused on ZBTUO combination therapy. Furthermore, the dose of CCPPs was 0.6 g/time or 1.2 g/time for XZK, 1.05 g/time for ZBTUO, and 0.24 g/time or 0.48 g/time for ZBTAI. Notably, the duration of treatment varies from 1 to 6 months, with XZK treatment typically administered for 3 months, and ZBTAI or ZBTUO treatment administered for 2 months. In the control group, atorvastatin, fluvastatin, pitavastatin, simvastatin, lovastatin, and rosuvastatin were mainly administered during statin therapy.

**TABLE 1:** Detailed information about the studies included.

Study	Subtypes of disease	Sample		Gender (M/F)		Age distribution		Treatment group	Control group	Study duration	Outcomes
		T	C	T	C	T	C				
Zou (2017)	dyslipidemia	244	244	134/110	140/104	52.6 ± 5.4	53.1 ± 5.5	XZK 0.6 g/bid + C	Atorvastatin 20 mg/qd	3 months	1,2,3,4,5,6
Shi et al. (2018)	dyslipidemia	62	62	34/28	35/27	62.13 ± 7.27	63.03 ± 7.52	XZK 0.6 g/bid + C	Fluvastatin 40 mg/qd	2 months	1,2,3,4,5,6
Zhang et al. (2017)	dyslipidemia	40	40	26/14	29/11	53.34 ± 13.64	54.62 ± 12.42	XZK 0.6 g/bid + C	Atorvastatin 40 mg/qd	1 month	1,2,3,4
Yu (2021)	dyslipidemia	38	38	20/18	20/18	58.69 ± 6.44	58.46 ± 6.28	XZK 0.6 g/bid + C	Atorvastatin 10 mg/qd	3 months	1,2,3,4,6
Wang (2012)	dyslipidemia	39	39	NR	NR	NR	NR	XZK 0.6 g/bid + C	Atorvastatin 10 mg/qd	3 months	1,2,3,4,5,6
Liu et al. (2018)	dyslipidemia	52	52	29/23	27/25	58.7 ± 3.8	58.5 ± 4.1	XZK 0.6 g/bid + C	Atorvastatin 20 mg/qd	2 months	1,2,3,4,5,6
Tian et al. (2022)	dyslipidemia	30	30	17/13	12/18	63.49 ± 3.05	64.02 ± 3.11	XZK 0.6 g/bid + C	Pitavastatin 2–4 mg/qd	3 months	1,2,3,4,5
Zhang and Tang (2010)	dyslipidemia	182	180	89/93	88/92	57.2 ± 10.8	56.5 ± 11.4	XZK 0.6g/bid + C	Simvastatin 10 mg/qd	2 months	1,2,3,4,5,6
Sun et al. (2018)	dyslipidemia	48	48	28/20	27/21	63.34 ± 7.29	63.39 ± 7.64	XZK 1.2g/bid + C	Simvastatin 20 mg/qd	3 months	1,2,3,4,5
Fu (2017)	dyslipidemia	75	75	36/39	38/37	63.2 ± 9.3	61.8 ± 9.3	XZK 0.6g/bid + C	Atorvastatin 10 mg/qd	3 months	1,2,3,4,5,6
Ma and Deng. (2019)	hypertriglyceridemia	76	74	NR	NR	NR	NR	XZK 0.6g/bid + C	Atorvastatin 40 mg/qd	6 months	1,2,3,4,6
Jiang and Chen. (2012)	dyslipidemia	85	85	42/43	40/45	61.7 ± 6.9	61.1 ± 7.4	XZK 1.2g/bid + C	Simvastatin 10 mg/qd	3 months	1,3,4,5
Qu et al. (2020)	dyslipidemia	39	39	22/17	25/14	50.08 ± 4.19	51.08 ± 4.27	XZK 0.6g/bid + C	Pitavastatin 2–4 mg/qd	3 months	1,2,3,4
Zhou (2010)	dyslipidemia	39	39	21/18	22/17	52.4 ± 5.5	53.2 ± 4.9	XZK 1.2g/bid + C	Simvastatin 10 mg/qd	1 month	1,2,3,4,5
Su (2021)	dyslipidemia	50	50	36/14	30/20	54.1	60.2	ZBTUO 1.05g/bid + C	Atorvastatin 20 mg/qd	1 month	1,2,3,4,5,6
Feng (2015)	dyslipidemia	60	60	36/24	34/26	67.3 ± 5.8	66.8 ± 5.6	ZBTUO 1.05 g/bid + C	Atorvastatin 10 mg/qd	3 months	1,2,3,4,5
Zhang et al. (2013)	dyslipidemia	85	85	46/39	45/40	63.76 ± 10.32	64.02 ± 9.05	ZBTUO 1.05 g/bid + C	Atorvastatin 10 mg/qd	2 months	1,2,3,4,5
Ji and Yi (2011)	dyslipidemia	66	66	NR	NR	NR	NR	ZBTUO 1.05 g/bid + C	Lovastatin 20 mg/qd	2 months	1,2,3,4,5,6
Li (2023)	dyslipidemia	55	55	29/26	27/28	70.92 ± 5.53	70.64 ± 5.2	ZBTAI 0.24 g/bid + C	Rosuvastatin 5 mg/qd	3 months	1,2,3,4,5,6
Yuan et al. (2023)	dyslipidemia	29	29	14/15	13/16	52.46 ± 3.89	52.24 ± 3.75	ZBTAI 0.24 g/bid + C	Atorvastatin 20 mg/qd	2 months	1,2,3,4,6
Chen et al. (2022)	dyslipidemia	60	60	37/23	39/21	63.94 ± 6.89	64.27 ± 6.76	ZBTAI 0.24g/bid + Statin	Atorvastatin 20 mg/qd	2 months	1,2,3,4,6
Tan et al. (2021)	dyslipidemia	60	60	34/26	35/25	83.05 ± 1.52	84.25 ± 0.75	ZBTAI 0.24 g/bid + C	Rosuvastatin 20 mg/qd	1 month	1,2,3,4,6
Xiong (2019)	dyslipidemia	45	45	25/20	27/18	66.32 ± 2.21	66.28 ± 2.18	ZBTAI 0.48 g/bid + C	Simvastatin 20 mg/qd	2 months	1,2,3,4,6
Tan (2020)	dyslipidemia	30	30	20/10	22/8	61.5 ± 18.5	62.5 ± 19.5	ZBTAI 0.24 g/bid + C	Atorvastatin 10 mg/qd	2 months	1,2,3,4,6
Li et al. (2016)	dyslipidemia	60	60	30/30	34/26	67.1 ± 2.3	67.5 ± 2.4	ZBTAI 0.24 g/bid +C	Rosuvastatin 10 mg/qd	2 months	1,2,3,4
Liu ZK et al. (2019)	dyslipidemia	43	50	NR	NR	NR	NR	ZBTAI 0.24 g/bid + C	Rosuvastatin 10 mg/qd	2 months	1,2,3,4,5
He (2020)	dyslipidemia	80	80	45/35	48/32	54.86 ± 10.08	55.01 ± 11.32	ZBTAI 0.48g/bid + C	Pitavastatin 2 mg/qd	2 months	1,2,3,4
Ma and Feng. (2018)	dyslipidemia	29	28	NR	NR	NR	NR	ZBTAI 0.24 g/bid + C	Rosuvastatin 5 mg/qd	2 months	1,2,3,4
Chen et al. (2020)	dyslipidemia	63	63	31/32	33/30	70.1 + 7.6	71.2 ± 8.1	ZBTAI 0.24 g/bid + C	Atorvastatin 10 mg/qd	3 months	1,2,3,4,5
Wang and Chen (2015)	dyslipidemia	32	32	18/14	19/13	67.5 ± 5.2	67.2 ± 4.5	ZBTAI 0.24 g/bid + C	Atorvastatin 10 mg/qd	6 weeks	1,2,3,4
Chen et al. (2016)	dyslipidemia	42	42	28/14	26/16	62.5 ± 5.6	63.5 ± 6.2	ZBTAI 0.24 g/bid + C	Atorvastatin 10 mg/qd	2 months	1,2,3,4,5
Liu Q et al. (2019)	dyslipidemia	45	45	27/18	29/16	73.9 ± 8.2	72.7 ± 8.5	ZBTAI 0.48 g/bid + C	Rosuvastatin 10 mg/qd	6 months	1,2,3,4
Shi (2019)	dyslipidemia	65	65	31/34	32/33	52.63 ± 5.42	52.59 ± 5.39	ZBTAI 0.24 g/bid + C	Simvastatin 20 mg/qd	2 months	1,2,3,4,5

Abbreviations: C, control group; M/F, male/female; NR, not reported; T, treatment group; XZK, xuezhikang capsule; ZBTAI, zhibitai capsule; ZBTUO, zhibituo capsule; Outcomes 1: total cholesterol (TC); 2: triglyceride (TG); 3: high-density lipoprotein cholesterol (HDL-C); 4: low-density lipoprotein cholesterol (LDL-C); 5: clinical efficacy; 6:adverse reaction.

### 3.3 Risk of bias

**FIGURE 2:**
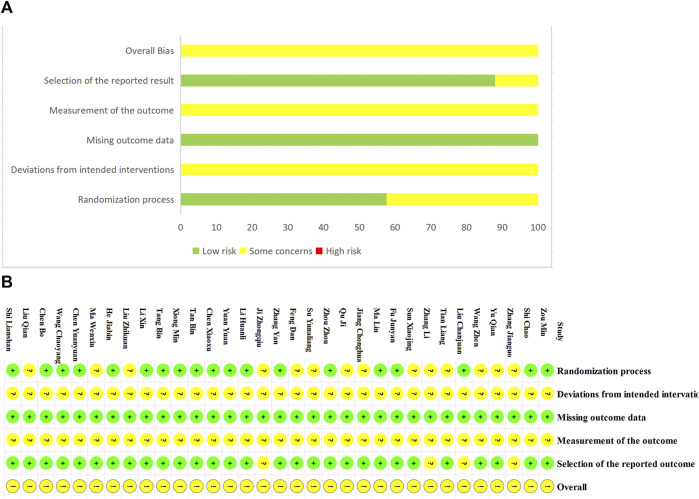
Risk of bias. **(A)** Risk of bias summary. **(B)** Risk of bias graph.

Nineteen RCTs (Zhou, 2010; Zhang et al., 2013; Wang and Chen, 2015; Chen et al., 2016; Li et al., 2016; Fu, 2017; Zou, 2017; Liu et al., 2018; Shi et al., 2018; Ma and Deng, 2019; Shi, 2019; Xiong, 2019; Chen et al., 2020; He, 2020; Tan, 2020; Tan et al., 2021; Chen et al., 2022; Li, 2023; Yuan et al., 2023) provided adequate randomization procedures and were assessed as low risk, while the others were deemed to have unclear risks due to the lack of specific details regarding randomization. Since none of the studies reported the information of allocation concealment, blinding, and measurement of the outcome, they were rated as unclear. All studies published complete data regarding the outcomes and were assessed as low risk. In addition, the selection of the reported results by the four studies (Zhang and Tang, 2010; Ji and Yi, 2011; Zhang et al., 2017; Liu et al., 2018) was concerning and assessed as unclear (Figure 2).

### 3.4 Outcomes measures

#### 3.4.1 Total cholesterol (TC)

**FIGURE 3:**
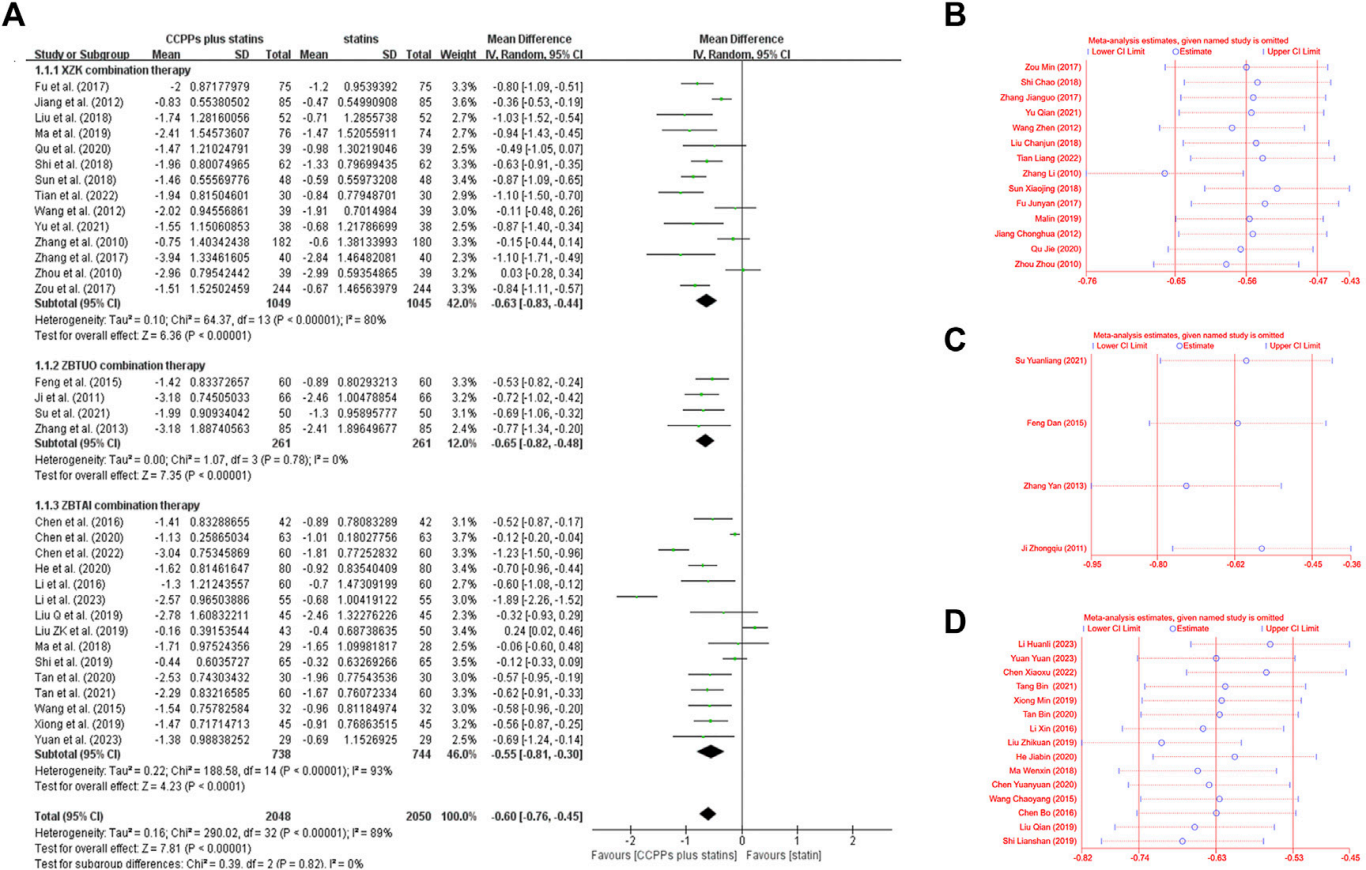
Effect of RYR-containing CCPPs on TC. **(A)** Forest plot of TC. **(B)** Sensitivity analysis of XZK on TC. **(C)** Sensitivity analysis of ZBTUO on TC. **(D)** Sensitivity analysis of ZBTAI on TC.

Total cholesterol levels were reported in all trials, of which 14 trials focused on the XZK combination therapy, 15 trials focused on the ZBTAI combination therapy, and 4 trials focused on the ZBTUO combination therapy. Random effect model was chosen because of the strong heterogeneity (*I* ^*2*^ = 89%, *p* < 0.0001). The results showed that treatment with RYR-containing CCPPs resulted in greater reductions in TC levels compared to that with statin [MD: 0.60, 95%CI(–0.76, −0.45), *p* < 0.00001], regardless of whether the patients were in XZK combination therapy group [MD: 0.63, 95%CI(–0.83, −0.44), *p* < 0.00001, *I* ^*2*^ = 80%], ZBTAI combination therapy group [MD: 0.55, 95%CI(–0.8, −0.30), *p* < 0.00001, *I* ^*2*^ = 93%], or ZBTUO combination therapy group [MD: 0.65, 95%CI(–0.82, −0.48), *p* < 0.00001, *I* ^*2*^ = 0%] (Figure 3A). The results of sensitivity analyses revealed that the overall values of the analysis were consistent with each other, the conclusions were reliable (Figures 3B–D).

**FIGURE 4:**
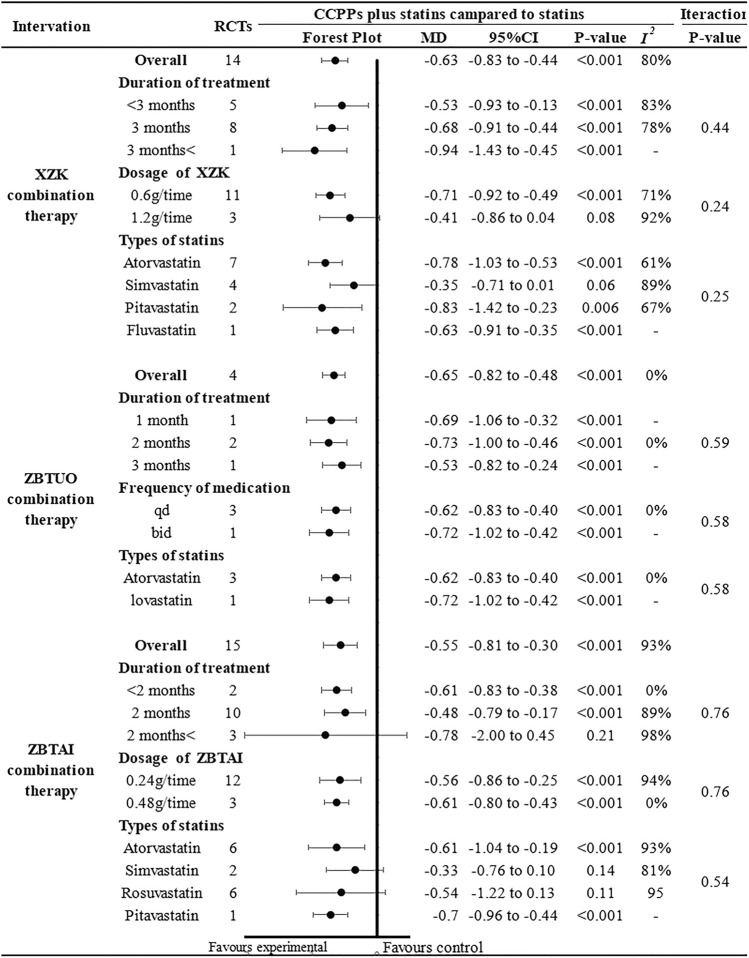
Subgroup analysis of the TC.

Further subgroup analysis was performed based on the CCPP dose, medication frequency, treatment duration, and statin type to investigate the influence of these parameters on the therapeutic effect of RYR-containing CCPPs (Figure 4). In case of ZBTUO combination therapy, the results for all the subgroups were similar to the overall conclusions. Moreover, treatment duration did not affect XZK efficacy, and the CCPP dose did not affect ZBTAI efficacy. However, XZK combination therapy did not result in significant TC reduction when the dose of XZK was 1.2 g/time [MD: 0.41, 95%CI(–0.86, 0.04), *p* = 0.08] or when XZK was combined with simvastatin [MD: 0.35, 95%CI(–0.71, 0.01), *p* = 0.06]. In the case of ZBTAI combination therapy, there was no significant positive effect in reducing TC reduction when the treatment duration exceeded 2 months [MD: 0.78, 95%CI(–2.00, 0.45), *p* = 0.21], or when ZBTAI was used in combination with simvastatin [MD: 0.33, 95%CI(–0.76, 0.10), *p* = 0.14] or rosuvastatin [MD: 0.54, 95%CI(–1.22, 0.13), *p* = 0.11].

**FIGURE 5:**
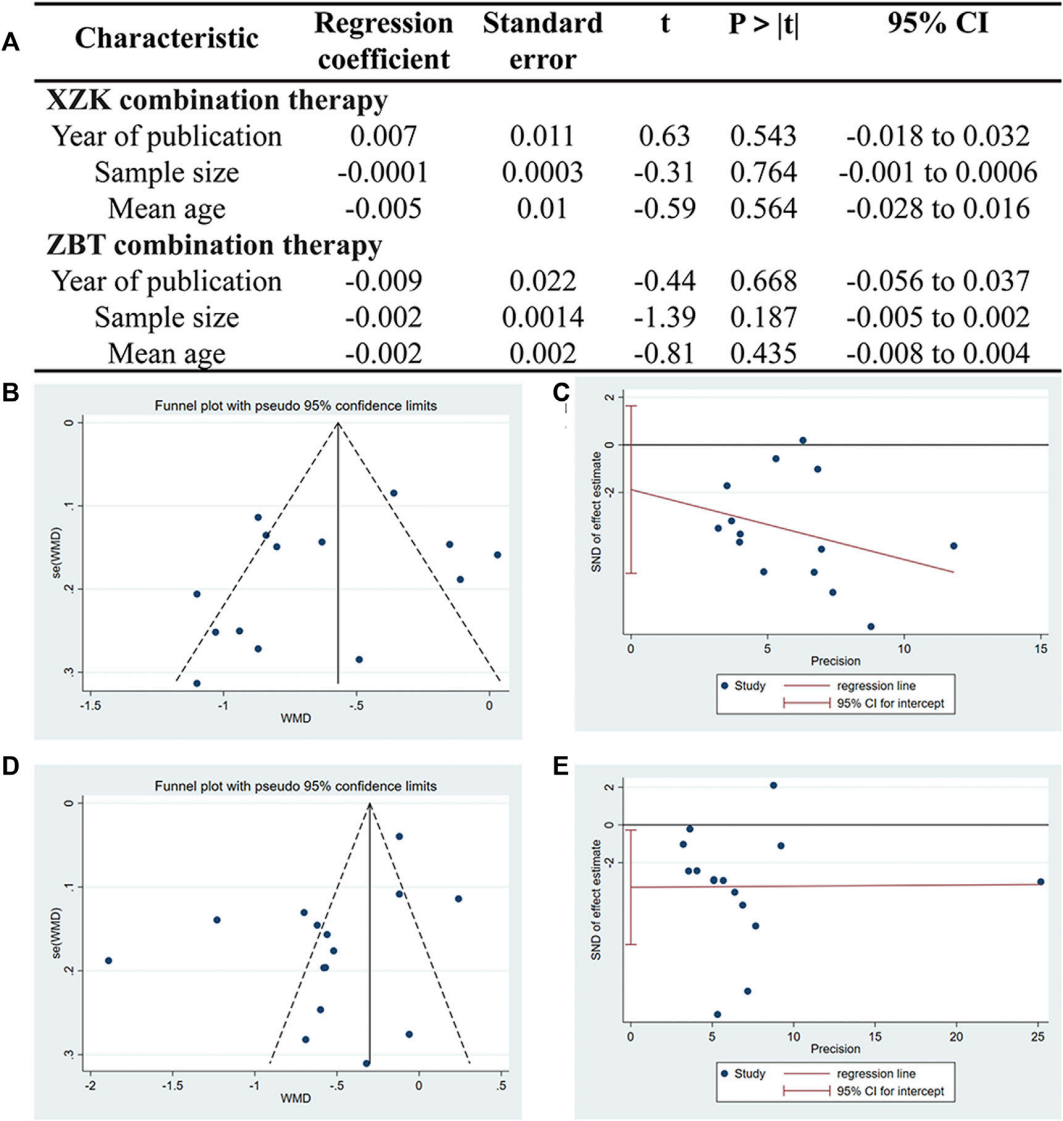
**(A)** Meta-regression was used to conduct the sources of heterogeneity. **(B)** Funnel plots of XZK combination therapy. **(C)** Egger’s test quantified the publication bias of XZK combination therapy. **(D)** Funnel plots of ZBTAI combination therapy. **(E)** Egger’s test quantified the publication bias of ZBTAI combination therapy.

Given the heterogeneity in both XZK combination therapies and ZBTAI combination therapies, meta-regression was performed to investigate the source of heterogeneity. Notable, no linear relationships were identified between variables and the outcome indicators, suggesting that these variables were not the source of heterogeneity (Figure 5A). Furthermore, the funnel plot and Egger’s test (P_XZK_ *=* 0.267) revealed no significant publication bias in XZK combination therapy (Figures 5B, C). However, the asymmetry of the funnel plot and the results of the Egger’s test (P_ZBTAI_ *=* 0.035) performed the presence of publication bias (Figures 5D, E) in ZBTAI combination therapy. Furthermore, trim-and-fill test was performed to evaluate the influence of the publication bias on the explanation of the results; and the results suggested that some studied showing negative findings was unpublished, which could influence the conclusions (Supplementary Table S6; Supplementary Figure S1).

#### 3.4.2 Low-density lipoprotein cholesterol (LDL-C)

**FIGURE 6:**
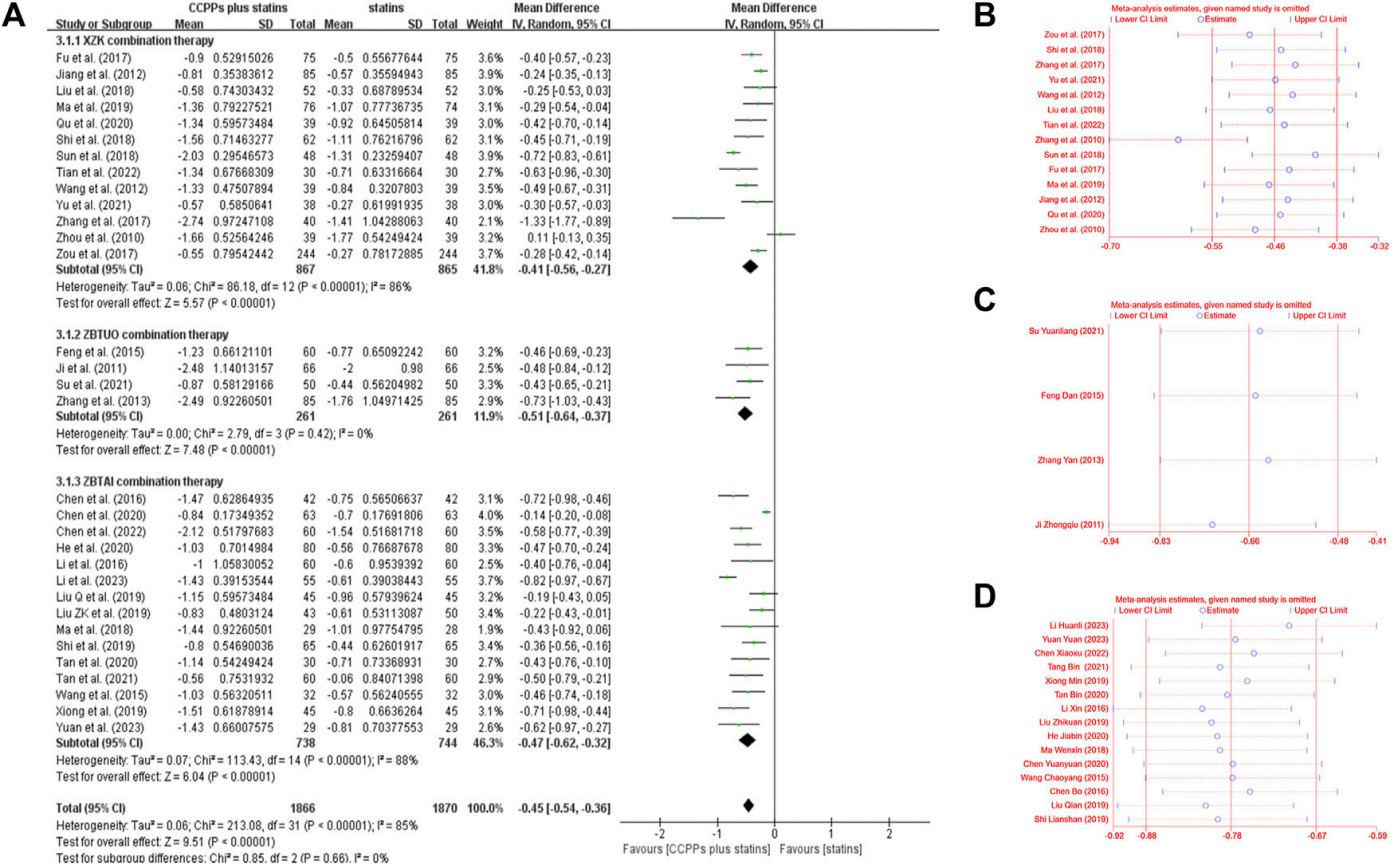
Effect of RYR-containing CCPPs on LDL-C. **(A)** Forest plot of LDL-C. **(B)** Sensitivity analysis of XZK on LDL-C. **(C)** Sensitivity analysis of ZBTUO on LDL-C. **(D)** Sensitivity analysis of ZBTAI on LDL-C.

Thirty-two RCTs involving 4,098 patients reported LDL-C levels, of which 13 trials focused on the XZK combination therapy, 15 trials focused on the ZBTAI combination therapy, and 4 trials focused on the ZBTUO combination therapy. Random effect model was chosen because of the strong heterogeneity (*I* ^*2*^ = 86%, *p* < 0.0001). The results showed that RYR-containing CCPPs resulted in greater reductions in the LDL-C levels compared to statin [MD: 0.45, 95%CI(–0.54, −0.36), *p* < 0.00001], regardless of whether the patients were in XZK combination therapy group [MD: 0.37, 95%CI(–0.52, −0.22), *p* < 0.00001, *I* ^*2*^ = 88%], ZBTAI combination therapy group [MD = −0.47, 95%CI(–0.62, −0.32), *p* < 0.00001, *I* ^*2*^ = 88%], or ZBTUO combination therapy group [MD: 0.51, 95%CI(–0.64, −0.37), *p* < 0.00001, *I* ^*2*^ = 0%], respectively (Figure 6A). Sensitivity analyses showed that the conclusions were reliable (Figures 6B–D).

**FIGURE 7:**
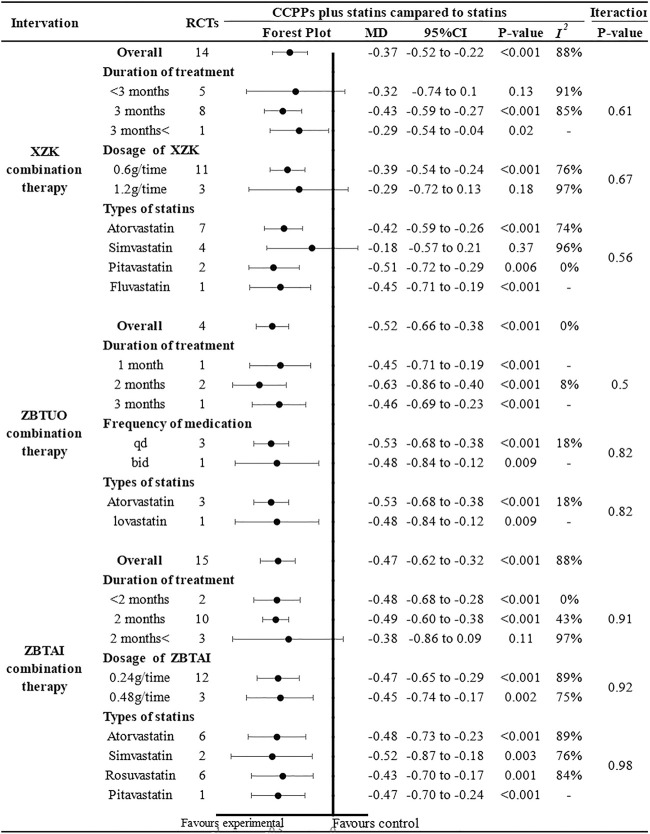
Subgroup analysis of the LDL-C.

The results of subgroup analyses showed that most subgroups were consistent with the overall findings, suggesting that most parameters did not significantly affect the notable efficacy of LDL-C reduction by RYR-containing CCPPs (Figure 7). However, when the dose of XZK was 1.2g/time [MD: 0.29, 95%CI(–0.72, 0.13), *p* = 0.18], the treatment duration was less than 3 months [MD: 0.32, 95%CI(–0.74, 0.1), *p* = 0.13] or XZK combined with simvastatin [MD: 0.18, 95%CI(–0.57, 0.21), *p* = 0.37], the XZK combination therapy did not show a significant positive effect in reducing LDL-C levels. Furthermore, in case of ZBTAI combination therapy, there was no significant positive effect on reducing LDL-C when the treatment duration exceeded 2 months [MD: 0.38, 95%CI(–0.86, 0.09), *p* = 0.11].

Given the heterogeneity in ZBTAI combination therapy, the meta-regression was conducted and not found the sources of heterogeneity (Supplementary Table S7). Additionally, the funnel plot and Egger’s test (P_XZK_ *=* 0.701) revealed that there was no significant publication bias in XZK combination therapy (Supplementary Figure S2). However, publication bias was found in ZBTAI combination therapy, and the results of trim-and-fill test suggested that publication bias could influence the conclusion (Supplementary Table S8; Supplementary Figure S3).

#### 3.4.3 Triglyceride (TG)

**FIGURE 8:**
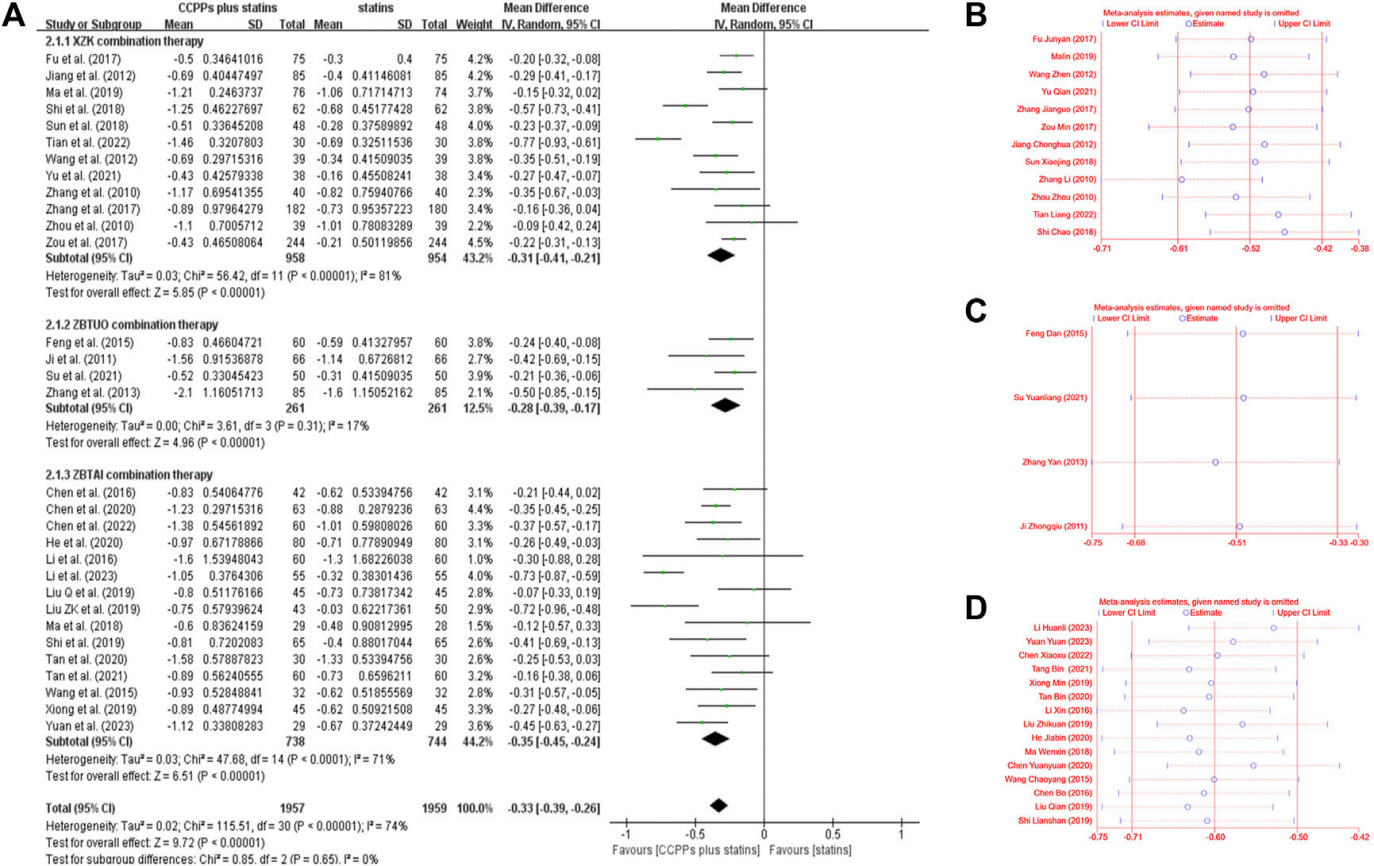
Effect of RYR-containing CCPPs on TG. **(A)** Forest plot of TG. **(B)** Sensitivity analysis of XZK on TG. **(C)** Sensitivity analysis of ZBTUO on TG. **(D)** Sensitivity analysis of ZBTAI on TG.

Thirty-one RCTs involving 3,816 patients reported the triglyceride, of which 12 trials focused on the XZK combination therapy, 15 trials focused on the ZBTAI combination therapy, and 4 trials focused on the ZBTUO combination therapy. Random effect model was chosen because of the strong heterogeneity (*I* ^*2*^ = 74%, *p* < 0.0001). The results showed that RYR-containing CCPPs resulted in greater a reduction in TG levels compared to statin [MD: 0.33, 95%CI(–0.39, −0.26), *p* < 0.00001], regardless of whether the patients were in XZK combination therapy group [MD: 0.31, 95%CI(–0.41, −0.21), *p* < 0.00001, *I* ^*2*^ = 81%], ZBTAI combination therapy group [MD: 0.35, 95%CI(–0.45, −0.24), *p* < 0.00001, *I* ^*2*^ = 71%], or ZBTUO combination therapy group [MD: 0.28, 95%CI(–0.39, −0.17, *p* < 0.00001), *I* ^*2*^ = 17%] (Figure 8A). Sensitivity analyses showed that the conclusions were reliable (Figures 8B–D).

**FIGURE 9:**
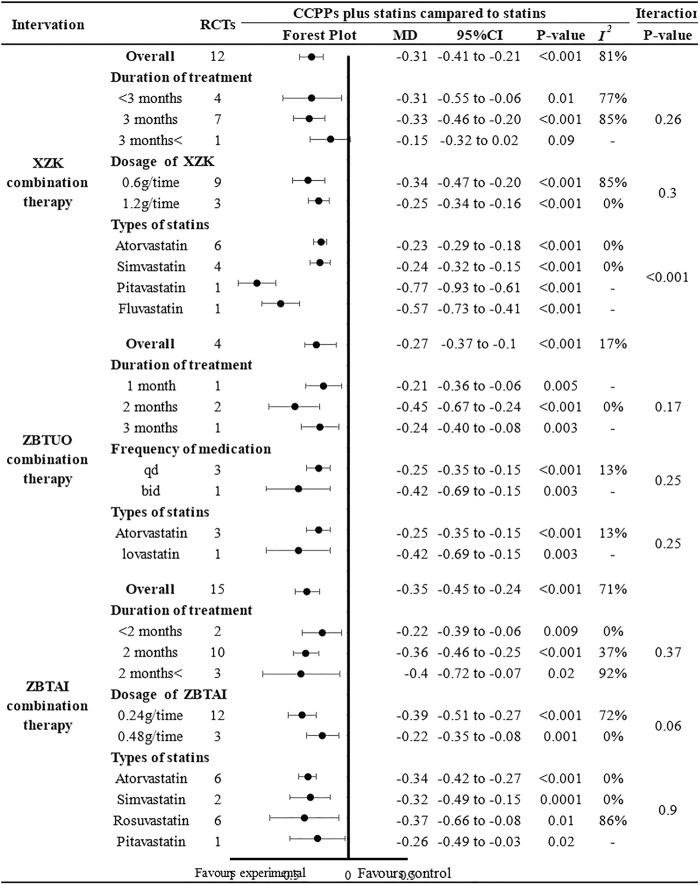
Subgroup analysis of the TG.

Further subgroup analyses were conducted and the results of most subgroups were consistent with the overall findings, suggesting that these parameters did not significantly influence the effect of both ZBTUO and ZBTAI on reducing the TG levels (Figure 9). However, in XZK combination therapy, there was no significant positive effect in reducing TG when the treatment duration exceeded 3 months [MD: 0.15, 95%CI(–0.32, 0.02), *p* = 0.09]. Notably, the high heterogeneity observed in XZK combination therapy was significantly reduced when subgroup analyses were conducted based on the statins types, suggesting that the type of statins was the source of heterogeneity. Furthermore, the difference in interaction effect among these subgroups of XZK combination therapy was highly significant (*P*_*iteration*_ < 0.001) when the subgroup analyses were conducted based on the types of statins, and these results indicate that the combination of XZK with pitavastatin would be optimal for therapy.

Given the heterogeneity in ZBTAI combination therapy, meta-regression was conducted and not found the sources of heterogeneity (Supplementary Table S9). Furthermore, the funnel plot and Egger’s test (P_XZK_ *=* 0.623, P_ZBTAI_ *=* 0.253) revealed that there was no significant publication bias in both XZK and ZBTAI (Supplementary Figure S4).

#### 3.4.4 High-density lipoprotein cholesterol (HDL-C)

**FIGURE 10:**
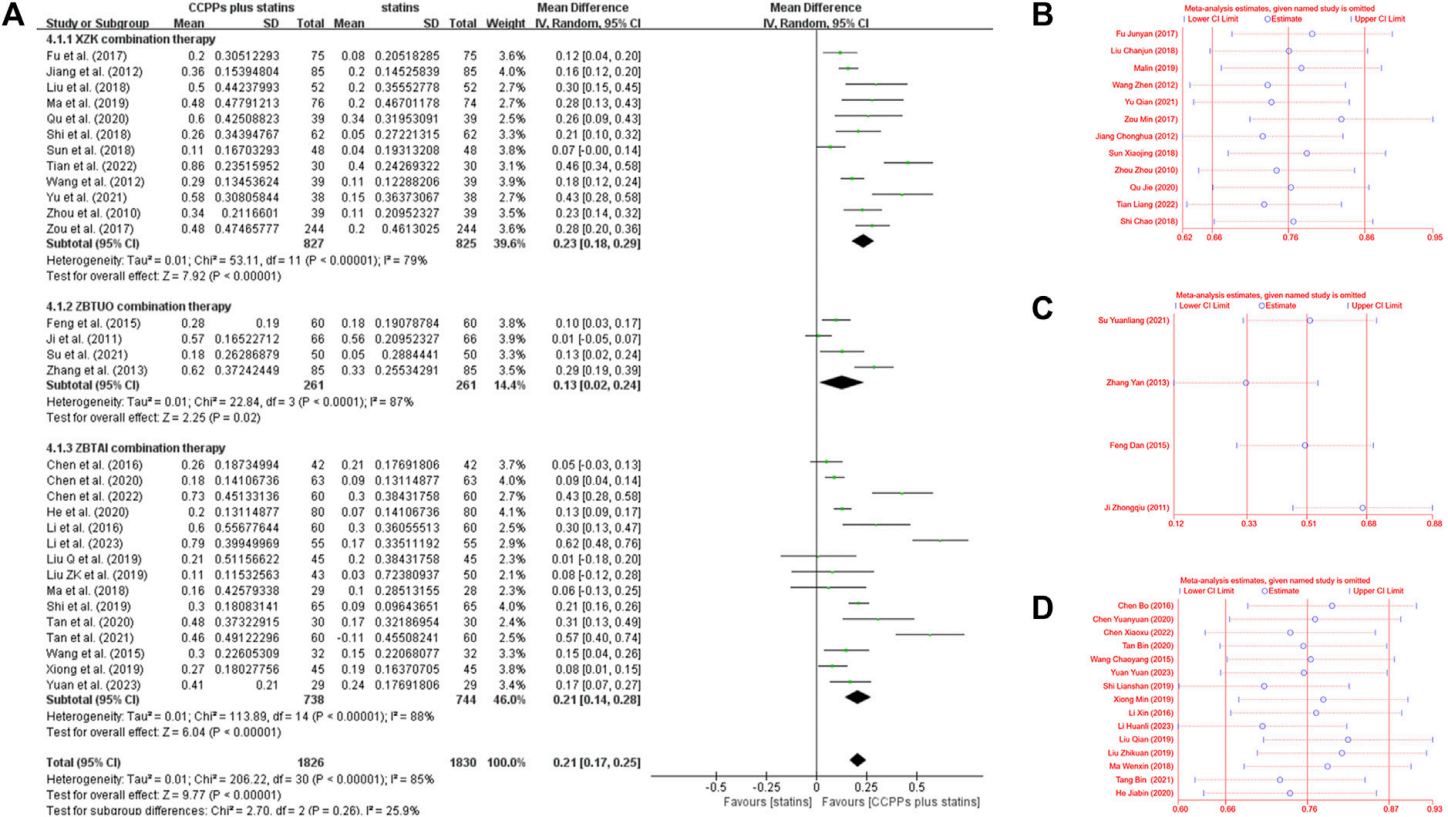
Effect of RYR-containing CCPPs on HDL-C. **(A)** Forest plot of HDL-C. **(B)** Sensitivity analysis of XZK on HDL-C. **(C)** Sensitivity analysis of ZBTUO on HDL-C. **(D)** Sensitivity analysis of ZBTAI on HDL-C.

Thirty-one RCTs reported the HDL-C levels, of which 12 trials focused on the XZK combination therapy, 15 trials focused on the ZBTAI combination therapy, and 4 trials focused on the ZBTUO combination therapy. Random effect model was chosen because of the strong heterogeneity (*I* ^*2*^ = 88%, *p* < 0.0001). The results showed that RYR-containing CCPPs resulted in greater improvements in HDL-C compared to statin [MD:0.21, 95%CI(0.17, 0.25), *p* < 0.00001], regardless of whether the patients were in XZK combination therapy group [MD:0.23, 95%CI(0.18, 0.29), *p* < 0.00001, *I* ^*2*^ = 79%], ZBTAI combination therapy group [MD:0.21, 95%CI(0.14, 0.28), *p* < 0.00001, *I* ^*2*^ = 88%], or ZBTUO combination therapy group [MD:0.13, 95%CI(0.02, 0.24), *p* < 0.00001, *I* ^*2*^ = 87%] (Figure 10A). Sensitivity analyses showed that the conclusions were reliable (Figures 10B–D).

**FIGURE 11:**
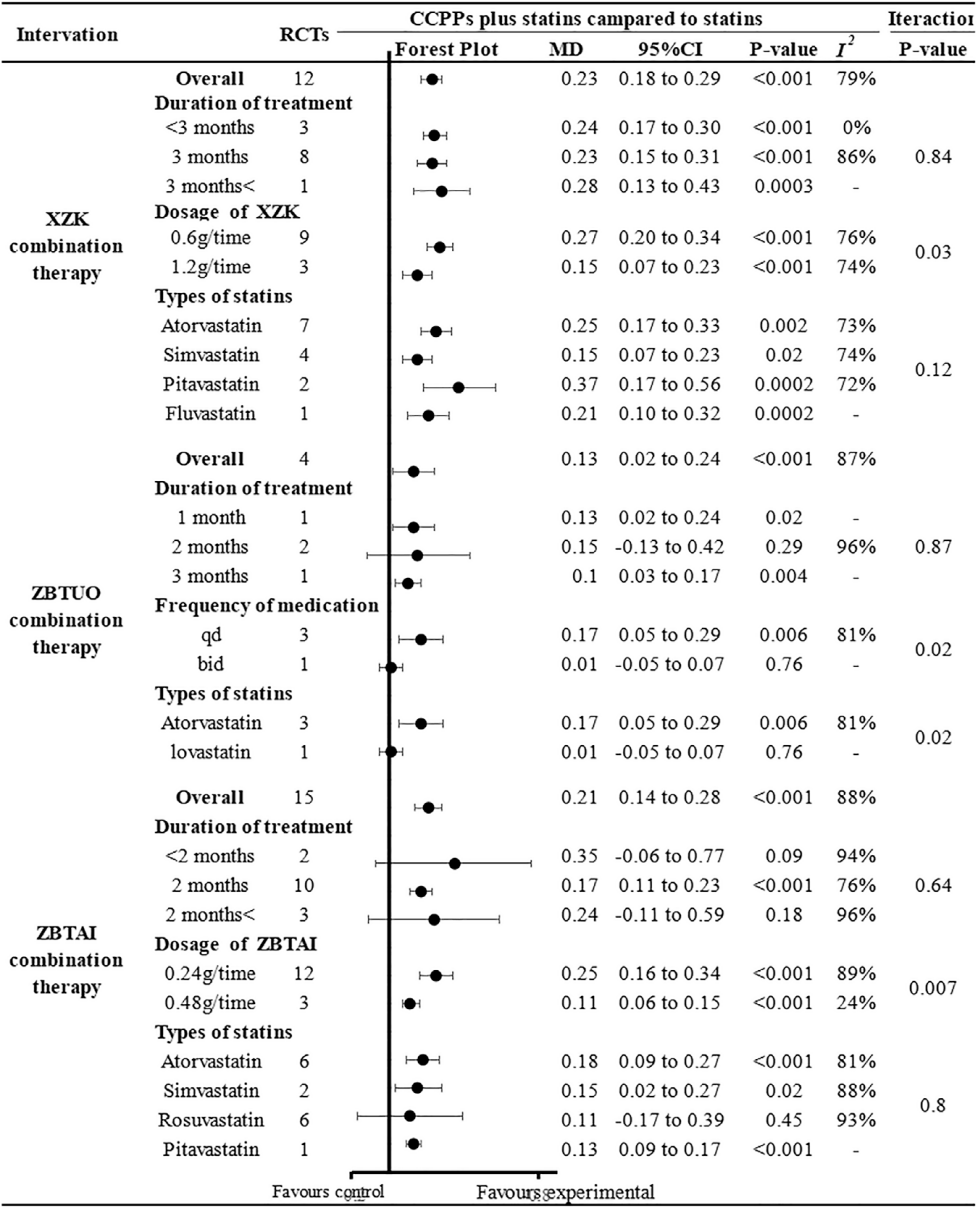
Subgroup analysis of the HDL-C.

Further subgroup analyses were performed to investigate the potential effects of specific parameters on the efficacy of RYR-containing CCPPs in improving HDL-C (Figure 11). (1) The results of all subgroups receiving XZK combination therapy were consistent with the overall findings. Additionally, the differences in interaction-related effects between these subgroups was highly significant (*P*_*iteration*_ < 0.05) when subgroup analyses were conducted based on the XZK dose, and these results indicated an optimal treatment dose of 0.6 g/time. (2) As for ZBTAI combination therapy, it was significantly effective in improving HDL-C levels only when the treatment duration was 2 months [MD:0.17, 95%CI(0.11, 0.23), *p* < 0.00001]. Furthermore, there was no significant effect when ZBTAI combined with rosuvastatin [MD:0.11, 95%CI(–0.17, 0.39), *p* = 0.45]. It is worth noting that the difference in interaction effect between subgroups indicate an optimal treatment dose of 0.24g/time. (3) Furthermore, the differences in interaction-related effects between subgroups receiving ZBTUO combination therapy were highly significant (*P*_*iteration*_<0.001) when subgroup analyses were based on the statins types or medication frequency. Furthermore, there was no significant positive effect of ZBTUO in reducing HDL-C levels when the medication frequency was bid [MD:0.01, 95%CI(–0.05, 0.07), *p* = 0.76], treatment duration was 2 months [MD:0.15, 95%CI(–0.13, 0.42), *p* = 0.29], or ZBTUO was combined with lovastatin [MD:0.01, 95%CI(–0.05, 0.07), *p* = 0.76].

Given the heterogeneity in ZBTAI combination therapy, ZBTUO combination therapy, and XZK combination therapy, the meta-regression was conducted, but sources of heterogeneity could not be identified (Supplementary Table S10). Additionally, the funnel plot and Egger’s test (P_ZBTAI_ *=* 0.115) suggested that there was no significant publication bias in ZBTAI combination therapy (Supplementary Figure S5). However, publication bias was found in XZK combination therapy, and the result of trim-and-fill test suggested that publication bias could influence the conclusions (Supplementary Table S11; Supplementary Figure S6).

#### 3.4.5 Clinical efficacy

**FIGURE 12:**
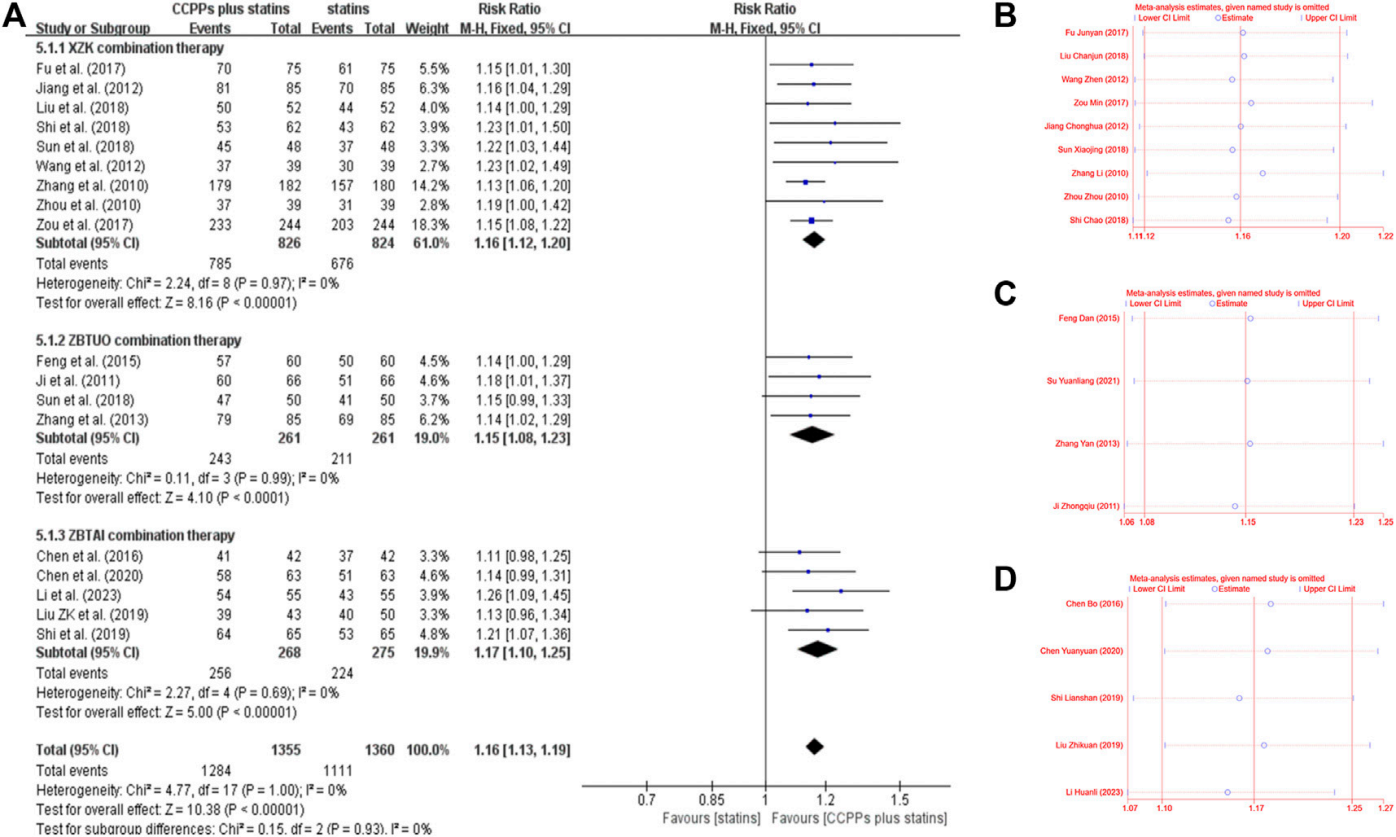
Effect of RYR-containing CCPPs on clinical efficacy. **(A)** Forest plot of clinical efficacy. **(B)** Sensitivity analysis of XZK on clinical efficacy. **(C)** Sensitivity analysis of ZBTUO on clinical efficacy. **(D)** Sensitivity analysis of ZBTAI on clinical efficacy.

Eighteen RCTs reported the clinical efficacy, of which 9 trials focused on the XZK combination therapy, 5 trials focused on the ZBTAI combination therapy, and 4 trials focused on the ZBTUO combination therapy. Fandom effect model was chosen because of the low heterogeneity (*I* ^*2*^ = 0%, *p* = 0.99). The results showed that the RYR-containing CCPPs was superior to statin [RR:1.16, 95%CI(1.12 to 1,20, *p* < 0.00001, *I* ^*2*^ = 0%], regardless of whether the patients were in XZK combination therapy group [RR:1.16, 95%CI(1.13, 1.19), *p* < 0.00001], ZBTAI combination therapy group [RR:1.17, 95%CI(1.10, 1.25), *p* < 0.00001, *I* ^*2*^ = 0%], or ZBTUO combination therapy group [RR:1.15, 95%CI(1.08, 1.23), *p* < 0.00001, *I* ^*2*^ = 0%] (Figure 12A). Sensitivity analyses showed that the conclusions were reliable (Figures 12B–D).

**FIGURE 13:**
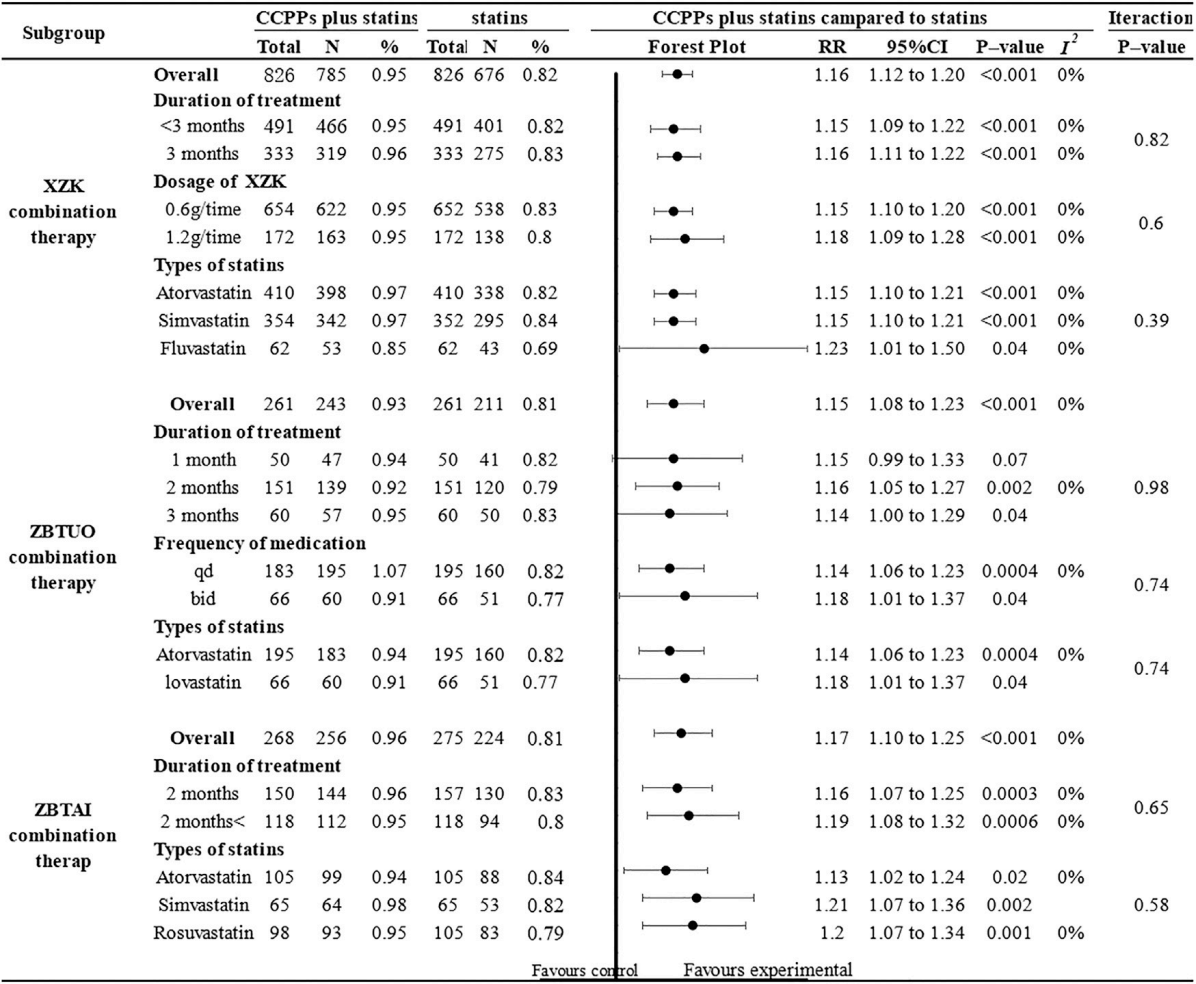
Subgroup analysis of the clinical efficacy.

Further subgroup analyses were conducted (Figure 13) and the results suggested that most subgroups were similar to the overall conclusions, indicating that the medication frequency, CCPP dose, and statin types did not significantly impact the efficacy of RYR-containing CCPPs. However, ZBTUO combination therapy did not show a significant positive effect at 1 month [RR:1.15, 95%CI(0.99, 1.33), *p* = 0.07] when considering the duration of treatment.

### 3.5 Adverse reactions

**FIGURE 14:**
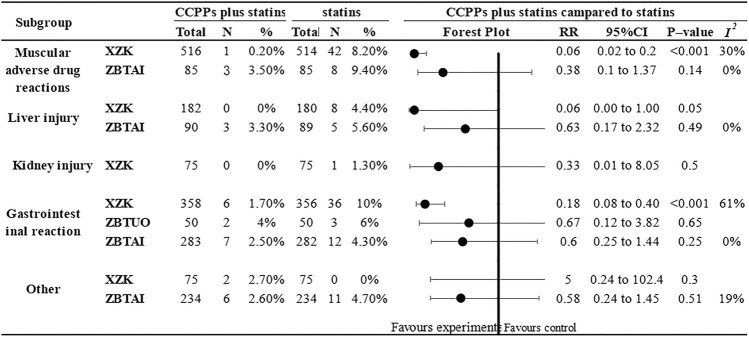
Subgroup analysis of the adverse reaction.

Eighteen RCTs reported adverse reactions. of which 8 trials focused on the XZK combination therapy, 9 trials focused on the ZBTAI combination therapy, and 1 trials focused on the ZBTUO combination therapy. Subgroup analysis (Figure 14) based on the type of adverse reaction demonstrated that XZK combination therapy was significantly better than statins in reducing the muscular adverse drug reactions [RR:0.06, 95%CI(0.02, 0.2), *p* < 0.00001, *I* ^*2*^ = 30%] and gastrointestinal reactions [RR:0.18, 95%CI(0.08, 0.40), *p* < 0.00001, *I* ^*2*^ = 62%]. However, there was no significant difference between the XZK and statins in reducing liver injury. Similarly, the results indicated that there was no significant difference between the ZBTAI and statins in reducing the muscular adverse drug reactions, gastrointestinal reactions, and liver injuries. Additionally, there was no significant difference between the ZBTUO and statins in reducing the kidney injuries. Notably, for other adverse reactions such as dizziness, headache, palpitations and rashes, no significant differences were observed between the treatment group and statin group. (Details were shown in Supplementary Table S12).

### 3.6 Quality of evidence

The quality of outcomes were evaluated by GRADE system (Supplementary Table S13). The results showed high-quality evidence for clinical efficacy in XZK combination therapy. In addition, moderate-quality evidence was obtained with two outcome indicators (TC, LDL-C) in XZK combination therapy, three outcome indicators (TG, HDL-C, and clinical efficacy) in ZBTAI combination therapy, and four outcome indicators (TC, TG, HDL-C, and clinical efficacy) in ZBTUO combination therapy. The remaining five outcome indicators were rated as low-quality evidence. The reasons for reducing the quality of evidence included publication bias, heterogeneity among studies, and number of included RCTs.

### 3.7 Evaluation of SR

AMSTAR-2 and ROBIS were used to assess the methodological quality and risk of bias of this meta analysis by two investigators (ZP and MYY) who did not have conflict of interest with this research. The results (Supplementary Tables S14, S15) confirmed that the risk of bias was low, and there were no significant methodological errors.

## 4 Discussion

### 4.1 Summary of evidence

Dyslipidemia, particularly an elevated LDL-C level, is a pathogenic risk factor for ASCVD that results in disease burden on patients and significant economic implications on the nation (Mach et al., 2020). In light of existing medical management strategies, there is an urgent need to further explore and evaluate treatment modalities. This study conducted a assessment of the efficacy of RYR-containing CCPPs combined with statins for treating dyslipidemia. A total of 33 trials involving 4,098 dyslipidemia patients were included. The results demonstrated that RYR-containing CCPPs had a substantial impact on increasing HDL-C levels and clinical efficacies, and decreasing TC, TG, and LDL-C levels, regardless of whether the administration of XZK, ZBTAI, or ZBTUO combination therapies. However, apart from the clinical efficacies, all the other results mentioned above exhibit heterogeneity. Furthermore, publication bias diminished our confidence in these results. The European Food Safety Authority has issued an opinion regarding a causal relationship between RYR administration and plasma LDL-C level reduction (EFSA Panel on Dietetic ProductsNutrition and Allergies, 2011). In addition, pharmacological research has indicated that monacolins, a complex of substances and an active metabolite of red yeast rice, possess a lactone form that is structurally identical to lovastatin. Monacolin K exhibits hypocholesterolemic effects by effectively and reversibly inhibiting β-hydroxyβ-methylglutaryl coenzyme A reductase (HMG-CoA), which is a crucial enzyme responsible for catalyzing the rate-limiting step in cholesterol biosynthesis, in a manner similar to that of other statins (Younes et al., 2018; Cicero et al., 2023). Notably, despite their identical structure, monacolin K and lovastatin exhibit different pharmacokinetic profiles and bioavailabilities (Banach et al., 2022; Buzzelli et al., 2023).

Moreover, increasing evidence suggests that statins have multiple adverse reactions, including liver and kidney injury, gastrointestinal reactions, and muscular adverse drug reactions. Hence, additional therapeutic options are needed to reduce the occurrence of adverse reactions (Stroes et al., 2015). Evidence from this study reveals that combining ZBTAI or XZK with statins significantly reduces the incidence of gastrointestinal disturbances and muscular adverse drug reactions. Current research also indicates that RYR-containing CCPPs do not increase the occurrence of other adverse reactions. A previous study showed that RYR demonstrates excellent tolerability even in dyslipidemia patients intolerant to statins, and these conclusions are similar to the results of this study (Cicero et al., 2017). Notably, another study revealed no significant association between monacolin K administration and an increased risk of musculoskeletal disorders (Awad et al., 2017; Fogacci et al., 2019). Moreover, a study showed that RYR exhibited a good safety profile with regard to the incidence of liver abnormalities and kidney injury (Gerards et al., 2015).

### 4.2 Secondary findings

Although RYR-containing CCPPs are widely used in clinical practice due to their safety and reliable efficacy, the credibility of the evidence has been diminished due to the lack of clarity regarding the optimal dose and treatment duration as well as the lack of data on drug combinations, which have posed challenges for clinical drug use (Ma et al., 2022). Therefore, Preplanned subgroup analysis was conducted to investigate the impact of treatment duration, statin type, and CCPP dose on the efficacy of RYR-containing CCPPs. With regard to the XZK capsule, we found that the optimal dose for improving HDL-C levels was 0.6 g/time, which aligned with the recommendations outlined in Chinese lipid management guidelines (Management, 2023). Conversely, no positive effect was observed on the reduction of LDL-C and TC levels when XZK was administered at a dose of 1.2 g/time. Furthermore, we found the source of heterogeneity among TGs was attributed to the statin types, and identified that the combination of XZK with pivastatin yielded the best therapeutic outcomes for reducing TG levels. An optimal dose of 0.24 g/time of the ZBTAI capsule, another common red yeast rice containing CCPPs, was found to improve HDL-C levels. While the number and quality of included studies may affect the credibility of these conclusions, the results can provide new ideas and directions for clinical research.

Due to the concept of “discontinue medication as soon as you observe effects” in traditional Chinese medicine theory, a clear medication course is not outlined for most CCPPs. However, long-term medication burdens the liver and kidney and results in other adverse reactions. Hence, it was necessary to assess the treatment duration (Cao et al., 2022). In this study, subgroup analysis based on CCPP treatment durations revealed that the optimal duration for XZK combination therapy was 3 months. Notably, our findings also indicated ZBTAI had a significant effect on improving blood lipid levels when the duration of treatment was 2 months. However, the above conclusions still need to be treated with caution and further research is necessary to validate them.

### 4.3 Quality of evidence

Given the high levels of heterogeneity of outcome indicators, meta-regression tests were used to found the sources of heterogeneity. Despite diligent efforts to mitigate heterogeneity, some outcome measures still exhibit heterogeneity, prompting cautious interpretation of conclusions. Notable, sensitivity analysis suggested the robustness of existing findings. Moreover, a trim-and-fill analysis revealed that several RCTs with negative results were unpublished. Therefore, caution must be exercised as these negative trials have the potential to overturn our current conclusions upon publication. Our assessment of the quality of evidence for outcome indicators indicated that most indicators had at least one factor leading to a downgrade. Specifically, one, nine, and five outcome indicators were rated as “high”, “moderate”, and “low” in terms of quality of evidence, respectively.

### 4.4 Advantages and limitations

This study provides updated evidence and has several advantages over previous research. In terms of interventions, we evaluated the efficacy of different types of RYR-containing CCPPs used for dyslipidemia treatment. Data regarding adverse reactions, categorized as liver and kidney injury, gastrointestinal reactions, and muscular adverse drug reactions, provided comprehensive evidence for assessing the safety of RYR-containing CCPPs. Meanwhile, adequate subgroup analyses of RYR-containing CCPPs were performed according to the characteristics of included studies, such as treatment duration, CCPPs dose, and drug combinations, and provided reliable evidence regarding the efficacy estimates of CCPPs. Notably, trim-and-fill analysis was used to evaluate the influence of publication bias on result interpretation, and meta-regression analyses were used to identify the source of heterogeneity, while sensitivity analysis was used to confirm the robustness of conclusions. Finally, the GRADE approach was employed to assess the overall strength of the evidence for each outcome measure. ROBIS and AMSTAR-2 were used to evaluate this study, which enhanced the credibility of the results.

However, this study is associated with several weaknesses. First of all, although this SR conducted a comprehensive literature search, the included studies were all conducted in China, and most of the studies were small sample studies, which may lead to low efficiency of statistical test. Second, our study did not evaluate the long-term efficacy of CCPPs, which is an important aspect of clinical evaluation. Third, subgroup analyses were conducted to investigate the effects of different types of dyslipidemia on the efficacy of RYR-containing CCPPs. However, only one of the included studies identified the types of dyslipidemia, which hindered the further evaluation of efficacy. Fourth, the majority of the included RCTs did not report about allocation concealment and blinding, which could affect the accuracy and reliability of the analysis results. Finally, although sensitivity analyses confirmed the robustness of these conclusions, existing conclusions need to be treated with caution due to heterogeneity and publication bias. In particular, the trim-and-fill analysis showed that some RCTs with negative results were not published, which would affect the reliability of the study results.

### 4.5 Implications for practice

Several invaluable suggestions were proposed for future research based on the findings and limitations of this study. Given the inconsistent results of subgroup analyses, further investigations are needed to explore the optimal dose and duration. Additionally, this study did not outline definitive conclusions regarding the effect of disease subtypes on treatment efficacy, which could be clinically significant. Therefore, future studies should identify the types of dyslipidemia and investigate the most effective treatments for each subtype. Considering the high incidence of dyslipidemia, long-term efficacy should be included as an outcome indicator in future trials. Furthermore, a trim-and-fill analysis revealed that some unpublished studies with negative findings would potentially impact existing conclusions. Hence, it is crucial to avoid selective reporting bias in future studies. In terms of clinical study more large-sample, multi-center, long-period RCTs should be conducted, and strictly follow the Consolidated Standards of Reporting Trials (CONSORT) guidelines to standardize research reports and make research more transparent. Moreover, it is crucial to conduct reasonable sample size estimation and implement random allocation, allocation concealment, and blinding methods in future studies. In summary, due to the limitations of this study, the results should be interpreted with caution until further confirmation of well-designed RCTs.

## 5 Conclusion

The combination of red yeast rice-containing CCPPs with statins appears to improve lipid profiles and clinical efficacy in patients with dyslipidemia, and has certain safety. However, due to the poor quality of the included studies, and some studied showing negative findings was unpublished. The results should be interpreted with caution until further confirmation by rigorous designs RCTs.

## Data Availability

The original contributions presented in the study are included in the article/Supplementary Material, further inquiries can be directed to the corresponding authors.

## Abbreviations

AMSTAR-2, Modified Quality Assessment Scale for Systematic Reviews; ApoA1, apolipoprotein A1; ApoB, apolipoprotein B; ASCVD, arteriosclerotic cardiovascular disease; CCPP, commercial Chinese polyherbal preparation; CVD, cardiovascular disease; GRADE, Grading of Recommendation Assessment Development and Evaluation; HDL-C, high-density lipoprotein-cholesterol; LDL-C, low-density lipoprotein-cholesterol; MD, mean difference; M/F, male/female; PRISMA, referred Reporting Program for Systematic Review and Meta-Analysis; RCTs, randomized controlled trials; ROBIS, Risk of bias in systematic reviews tool; RYR, red yeast rice; RR, relative risk; SR, systematic review; TC, total cholesterol; TG, triglyceride; XZK, Xuezhikang capsule; ZBTAI, Zhibitai capsule; ZBTUO, Zhibituo capsule; 95%CI, 95% confidence interval.
