# Supplementary material for: Effects of the combination of red yeast rice-containing commercial Chinese polyherbal preparation with statins for dyslipidemia: a systematic review and meta-analysis
Source: Front Pharmacol. 2024 Jul 23;15:1398934. doi: 10.3389/fphar.2024.1398934 (PMC11300347; doi:10.3389/fphar.2024.1398934)

**Effects of the combination of red yeast rice-containing commercial Chinese polyherbal preparation with statins for dyslipidemia: A systematic review and meta-analysis**

Menglong Shi^a,b,1^, Tianye Sun^c,1^, Chenyao Zhang ^a,b,1^, Yucong Ma^a,b^, Bo Pang^a,b^, Lujia Cao^a,b,e^, Zhaochen Ji^a,b,d,e^, Fengwen Yang^a,b**^, Junhua Zhang^a,b,e*^

^a^ Tianjin University of Traditional Chinese Medicine, Tianjin 301617, China

^b^ Evidence-Based Medicine Center, Tianjin University of Traditional Chinese Medicine, Tianjin 301617, China

^c^ Dongfang Hospital, Beijing University of Chinese Medicine, Beijing 100071, China
^d^ College of Traditional Chinese Medicine, Tianjin University of Traditional Chinese Medicine, Tianjin 301617, China

^e^ Haihe Laboratory of Modern Chinese Medicine, Tianjin University of Traditional Chinese Medicine, Tianjin 301617, China

**Keywords:** red yeast rice, dyslipidaemia, Xuezhikang capsule, Zhibitai capsule, Zhibituo capsule, meta-analysis

## Abbreviations

AMSTAR-2, Modified Quality Assessment Scale for Systematic Reviews; ASCVD, arteriosclerotic cardiovascular disease; CCPPs, commercial Chinese polyherbal preparation; CVD, cardiovascular disease; GRADE, Grading of Recommendation, Assessment, Development, and Evaluation; HDL-C, high-density lipoprotein-cholesterol; LDL-C, low-density lipoprotein-cholesterol; MD, mean difference; PRISMA, referred Reporting Program for Systematic Review and Meta-Analysis; RCTs, [randomized controlled trials](https://www.sciencedirect.com/topics/medicine-and-dentistry/randomized-controlled-trial); ROBIS, Risk of bias in systematic reviews tool; RYR, red yeast rice; RR, relative risk; SR, systematic review; TC, total cholesterol; TG, triglyceride; XZK, Xuezhikang capsule; ZBTAI, Zhibitai capsule; ZBTUO, Zhibituo capsule; 95%CI, 95% confidence interval.

* Corresponding author at: State Key Laboratory of Component-Based Chinese Medicine, Tianjin University of Traditional Chinese Medicine, Tianjin 301617, China

** Co-corresponding author at: State Key Laboratory of Component-Based Chinese Medicine, Tianjin University of Traditional Chinese Medicine, Tianjin 301617, China

E-mail addresses: : zjhtcm@foxmail.com (JH. Zhang), 13682027022@163.com (FW. Yang), .

^1^These authors contributed equally.

**Supplementary Materials**

**catalogue**

[Table S1 PRISMA checklist of the meta-analysis. 1](#_Toc12042)

[Table S2 Botanical drugs included of RYR-containing CCPP. 6](#_Toc25970)

[Table S3 Search strategies. 7](#_Toc20162)

[Table S4 The criteria of Outcome indicators. 11](#_Toc30825)

[Table S5 The reasons of excluded 23studies. 12](#_Toc26917)

[Table S6 Trim-and-fill test of ZBTAI combination therapy in reducing TC. 15](#_Toc12940)

[Table S7 Meta-regression of ZBTAI combination therapy in reducing LDL-C. 16](#_Toc5492)

[Table S8 Trim-and-fill test of ZBTAI combination therapy in reducing LDL-C. 17](#_Toc14740)

[Table S9 Meta-regression of ZBTAI combination therapy in reducing TG. 18](#_Toc2073)

[Table S10 Meta-regression of red yeast rice-containing CCPPs in reducing HDL-C. 19](#_Toc18319)

[Table S11 Trim-and-fill test of XZK combination therapy in reducing HDL-C. 20](#_Toc13579)

[Table S12 Details of the adverse event occurred in the included studies. 21](#_Toc8752)

[Table S13 Summary of findings. 22](#_Toc20067)

[Table S14 Methodological quality assessment 25](#_Toc29080)

[Table S15 the risk of bias in this systematic review. 27](#_Toc18561)

[Fig. S1 Trim-and-fill test of ZBTAI combination therapy in reducing TC. 30](#_Toc6749)

[Fig. S2 Publication bias of red yeast rice-containing CCPPs on LDL-C. 30](#_Toc6260)

[Fig. S3 Trim-and-fill test of ZBTAI combination therapy in reducing LDL-C. 31](#_Toc18050)

[Fig. S4 Publication bias of red yeast rice-containing CCPPs on TG. 31](#_Toc19493)

[Fig. S5 Publication bias of red yeast rice-containing CCPPs on HDL-C. 31](#_Toc32252)

[Fig. S6 Trim-and-fill test of XZK combination therapy in reducing HDL-C. 32](#_Toc228)

**Table S1 PRISMA checklist of the meta-analysis.**

| **Section and Topic** | **Item #** | **Checklist item** | **Location where item is reported** |
| --- | --- | --- | --- |
| **TITLE** | | |  |
| Title | 1 | Identify the report as a systematic review. |  |
| **ABSTRACT** | | |  |
| Abstract | 2 | See the PRISMA 2020 for Abstracts checklist. |  |
| **INTRODUCTION** | | |  |
| Rationale | 3 | Describe the rationale for the review in the context of existing knowledge. |  |
| Objectives | 4 | Provide an explicit statement of the objective(s) or question(s) the review addresses. |  |
| **METHODS** | | |  |
| Eligibility criteria | 5 | Specify the inclusion and exclusion criteria for the review and how studies were grouped for the syntheses. |  |
| Information sources | 6 | Specify all databases, registers, websites, organisations, reference lists and other sources searched or consulted to identify studies. Specify the date when each source was last searched or consulted. |  |
| Search strategy | 7 | Present the full search strategies for all databases, registers and websites, including any filters and limits used. | Supplementary TableS3 |
| Selection process | 8 | Specify the methods used to decide whether a study met the inclusion criteria of the review, including how many reviewers screened each record and each report retrieved, whether they worked independently, and if applicable, details of automation tools used in the process. |  |
| Data collection process | 9 | Specify the methods used to collect data from reports, including how many reviewers collected data from each report, whether they worked independently, any processes for obtaining or confirming data from study investigators, and if applicable, details of automation tools used in the process. |  |
| Data items | 10a | List and define all outcomes for which data were sought. Specify whether all results that were compatible with each outcome domain in each study were sought (e.g. for all measures, time points, analyses), and if not, the methods used to decide which results to collect. |  |
|  | 10b | List and define all other variables for which data were sought (e.g. participant and intervention characteristics, funding sources). Describe any assumptions made about any missing or unclear information. |  |
| Study risk of bias assessment | 11 | Specify the methods used to assess risk of bias in the included studies, including details of the tool(s) used, how many reviewers assessed each study and whether they worked independently, and if applicable, details of automation tools used in the process. |  |
| Effect measures | 12 | Specify for each outcome the effect measure(s) (e.g. risk ratio, mean difference) used in the synthesis or presentation of results. |  |
| Synthesis methods | 13a | Describe the processes used to decide which studies were eligible for each synthesis (e.g. tabulating the study intervention characteristics and comparing against the planned groups for each synthesis (item #5)). |  |
|  | 13b | Describe any methods required to prepare the data for presentation or synthesis, such as handling of missing summary statistics, or data conversions. |  |
|  | 13c | Describe any methods used to tabulate or visually display results of individual studies and syntheses. |  |
|  | 13d | Describe any methods used to synthesize results and provide a rationale for the choice(s). If meta-analysis was performed, describe the model(s), method(s) to identify the presence and extent of statistical heterogeneity, and software package(s) used. |  |
|  | 13e | Describe any methods used to explore possible causes of heterogeneity among study results (e.g. subgroup analysis, meta-regression). |  |
|  | 13f | Describe any sensitivity analyses conducted to assess robustness of the synthesized results. |  |
| Reporting bias assessment | 14 | Describe any methods used to assess risk of bias due to missing results in a synthesis (arising from reporting biases). |  |
| Certainty assessment | 15 | Describe any methods used to assess certainty (or confidence) in the body of evidence for an outcome. |  |
| **RESULTS** | | |  |
| Study selection | 16a | Describe the results of the search and selection process, from the number of records identified in the search to the number of studies included in the review, ideally using a flow diagram. | Figure 1 |
|  | 16b | Cite studies that might appear to meet the inclusion criteria, but which were excluded, and explain why they were excluded. | Table S5 |
| Study characteristics | 17 | Cite each included study and present its characteristics. | Table1 |
| Risk of bias in studies | 18 | Present assessments of risk of bias for each included study. | Figure 2 |
| Results of individual studies | 19 | For all outcomes, present, for each study: (a) summary statistics for each group (where appropriate) and (b) an effect estimate and its precision (e.g. confidence/credible interval), ideally using structured tables or plots. |  |
| Results of syntheses | 20a | For each synthesis, briefly summarise the characteristics and risk of bias among contributing studies. |  |
|  | 20b | Present results of all statistical syntheses conducted. If meta-analysis was done, present for each the summary estimate and its precision (e.g. confidence/credible interval) and measures of statistical heterogeneity. If comparing groups, describe the direction of the effect. |  |
|  | 20c | Present results of all investigations of possible causes of heterogeneity among study results. |  |
|  | 20d | Present results of all sensitivity analyses conducted to assess the robustness of the synthesized results. |  |
| Reporting biases | 21 | Present assessments of risk of bias due to missing results (arising from reporting biases) for each synthesis assessed. |  |
| Certainty of evidence | 22 | Present assessments of certainty (or confidence) in the body of evidence for each outcome assessed. | Table2 |
| **DISCUSSION** | | |  |
| Discussion | 23a | Provide a general interpretation of the results in the context of other evidence. |  |
|  | 23b | Discuss any limitations of the evidence included in the review. |  |
|  | 23c | Discuss any limitations of the review processes used. |  |
|  | 23d | Discuss implications of the results for practice, policy, and future research. |  |
| **OTHER INFORMATION** | | |  |
| Registration and protocol | 24a | Provide registration information for the review, including register name and registration number, or state that the review was not registered. |  |
|  | 24b | Indicate where the review protocol can be accessed, or state that a protocol was not prepared. |  |
|  | 24c | Describe and explain any amendments to information provided at registration or in the protocol. |  |
| Support | 25 | Describe sources of financial or non-financial support for the review, and the role of the funders or sponsors in the review. |  |
| Competing interests | 26 | Declare any competing interests of review authors. |  |
| Availability of data, code and other materials | 27 | Report which of the following are publicly available and where they can be found: template data collection forms; data extracted from included studies; data used for all analyses; analytic code; any other materials used in the review. | Supplementary |

**Table S2 Botanical drugs included of RYR-containing CCPP.**

| **Abbreviation** | **Component** | **Traditional efficacy** |
| --- | --- | --- |
| Xuezhikang capsule | *red yeast rice (RYR) [Rice fermented by monascus]* | Removing dampness and expelling phlegm, promoting blood and removing stasis, strengthening spleen and eliminating food |
| Zhibitai capsule | *Crataegus pinnatifida Bunge (CPB) [Rosaceae;* [*crataegi fructus*](https://mpns.science.kew.org/mpns-portal/drugDetail?drugName=crataegi+fructus&query=Shanzha&filter=&fuzzy=false&nameType=all)*], Alisma plantago-aquatica subsp. Orientale (Sam.) Sam. (AR) [Alismataceae;* [*alismatis rhizoma*](https://mpns.science.kew.org/mpns-portal/drugDetail?drugName=alismatis+rhizoma&query=Zexie&filter=&fuzzy=false&nameType=all)*], Atractylodes macrocephala Koidz (AMR) [Asteraceae;* [*atractylodis macrocephalae rhizoma*](https://mpns.science.kew.org/mpns-portal/drugDetail?drugName=atractylodis+macrocephalae+rhizoma&query=Baizhu&filter=&fuzzy=false&nameType=all)*], and red yeast rice (RYR) [Rice fermented by monascus].* | Eliminating phlegm and removing blood stasis, strengthening spleen and stomach. |
| Zhibituo capsule | *Crataegus pinnatifida Bunge (CPB) [Rosaceae;* [*crataegi fructus*](https://mpns.science.kew.org/mpns-portal/drugDetail?drugName=crataegi+fructus&query=Shanzha&filter=&fuzzy=false&nameType=all)*], Alisma plantago-aquatica subsp, Atractylodes macrocephala Koidz (AMR) [Asteraceae;* [*atractylodis macrocephalae rhizoma*](https://mpns.science.kew.org/mpns-portal/drugDetail?drugName=atractylodis+macrocephalae+rhizoma&query=Baizhu&filter=&fuzzy=false&nameType=all)*], and red yeast rice (RYR) [Rice fermented by monascus].* | Eliminating phlegm and removing blood stasis, strengthening spleen and stomach. |

**Table S3 Search strategies.**

| **#** | **Searches** |
| --- | --- |
| **Search strategies of CNKI (Searched from inception to Nov. 22, 2023 and found 411 literature)** | |
| **#1** | TKA=('血脂异常'+'高脂血症'+'高血脂'+'高胆固醇血症'+'高甘油三酯血症'+'混合型高脂血症'+'低高密度脂蛋白血症'+'高脂蛋白血症'+'血脂过多'+'脂代谢异常')*('红曲'+'红曲米'+'血脂康'+'脂必泰'+'脂必妥')*('他汀'+'辛伐他汀'+'洛伐他汀'+'氟伐他汀'+'阿托伐他汀'+'匹伐他汀'+'普伐他汀'+'瑞舒伐他汀')*('随机对照试验'+'临床对照试验'+'随机'+'安慰剂'+'对照'+'盲法'+'试验'+'分组') |
| **Search strategies of Wanfang (Searched from inception to Nov. 22, 2023 and found 345 literature)** | |
| **#1** | 主题: (“血脂异常” or “高脂血症” or “高血脂” or “高胆固醇血症” or “高甘油三酯血症” or “混合型高脂血症” or “低高密度脂蛋白血症” or “高脂蛋白血症” or “血脂过多” or “脂代谢异常”) and (“红曲” or “红曲米” or “血脂康” or “脂必泰” or “脂必妥”) and (“他汀” or “辛伐他汀” or “洛伐他汀” or “氟伐他汀” or “阿托伐他汀” or “匹伐他汀” or “普伐他汀” or “瑞舒伐他汀”) and (“随机对照试验” or “临床对照试验” or “随机” or “安慰剂” or “对照” or “盲法” or “试验” or “分组”) |
| **Search strategies of VIP (Searched from inception to Nov. 22, 2023 and found 292 literature)** | |
| **#1** | M=(血脂异常 OR 高脂血症 OR 高血脂 OR 高胆固醇血症 OR 高甘油三酯血症 OR 混合型高脂血症 OR 低高密度脂蛋白血症 OR 高脂蛋白血症 OR 血脂过多 OR 脂代谢异常) AND M=(红曲 OR 红曲米 OR 血脂康 OR 脂必泰 OR 脂必妥) AND M=(他汀 OR 辛伐他汀 OR 洛伐他汀 OR 氟伐他汀 OR 阿托伐他汀 OR 匹伐他汀 OR 普伐他汀 OR 瑞舒伐他汀) AND R=(随机对照试验 OR 临床对照试验 OR 随机 OR 安慰剂 OR 盲法 OR 对照 OR 试验 OR 分组) |
| **Search strategies of SinoMed (Searched from inception to Nov. 22, 2023 and found 340 literature)** | |
| **#1** | ("血脂异常" OR "高脂血症" OR "高血脂" OR "高胆固醇血症" OR "高甘油三酯血症" OR "混合型高脂血症" OR "低高密度脂蛋白血症" OR "高脂蛋白血症" OR "血脂过多" OR "脂代谢异常") AND ("红曲" OR "红曲米" OR "血脂康" OR "脂必泰" OR "脂必妥") AND ("他汀" OR "辛伐他汀" OR "洛伐他汀" OR "氟伐他汀" OR "阿托伐他汀" OR "匹伐他汀" OR "普伐他汀" OR "瑞舒伐他汀") AND ("随机对照试验" OR "临床对照试验" OR "随机" OR "安慰剂" OR "盲法" OR "对照" OR "试验" OR "分组") |
| **Search strategies of PubMed (Searched from inception to Nov. 22, 2023 and found 61 literature)** | |
| **#1** | ((((((("Dyslipidemias"[Mesh])) OR "Hyperlipidemias"[Mesh]) OR "Hypercholesterolemia"[Mesh]) OR "Hyperlipidemia, Familial Combined"[Mesh]) OR "Hyperlipoproteinemias"[Mesh]) OR "Hypertriglyceridemia"[Mesh]) OR (((((((((((((((((((((Dyslipidemia[Title/Abstract]) OR (Dyslipoproteinemia*[Title/Abstract])) OR (Hyperlipemia*[Title/Abstract])) OR (Hyperlipidemia*[Title/Abstract])) OR (Lipidemia*[Title/Abstract])) OR (Lipemia*[Title/Abstract])) OR (Hypercholesterolemia*[Title/Abstract])) OR (High Cholesterol Level*[Title/Abstract])) OR (Cholesterol Level*, High[Title/Abstract])) OR (Level*, High Cholesterol[Title/Abstract])) OR (Elevated Cholesterol*[Title/Abstract])) OR (Cholesterol*, Elevated[Title/Abstract])) OR (Hypercholesteremia*[Title/Abstract])) OR (Hypertriglyceridemia*[Title/Abstract])) OR (Combined Hyperlipidemia*, Familial[Title/Abstract])) OR (Familial Combined Hyperlipidemia*[Title/Abstract])) OR (Hyperlipidemia*, Familial Combined[Title/Abstract])) OR (Hyperlipidemia*, Multiple Lipoprotein-Type[Title/Abstract])) OR (Lipoprotein-Type Hyperlipidemia*, Multiple[Title/Abstract])) OR (Multiple Lipoprotein-Type Hyperlipidemia*[Title/Abstract])) OR (Hyperlipoproteinemia*[Title/Abstract])) |
| **#2** | (((("Monascus"[Mesh]) OR ("red yeast rice" [Supplementary Concept])) OR (("xuezhikang" [Supplementary Concept]) OR "zhibitai" [Supplementary Concept])) OR (((((((((((Monascus purpureus[Title/Abstract]) OR (Monascus rubiginosus[Title/Abstract])) OR (Monascus araneosus[Title/Abstract])) OR (Monascus albidus[Title/Abstract])) OR (Monascus anka[Title/Abstract])) OR (Xuezhikang[Title/Abstract])) OR (Xuezhi Kang[Title/Abstract])) OR (Zhibitai[Title/Abstract])) OR (Zhibi Tai[Title/Abstract])) OR (Zhibituo[Title/Abstract])) OR (Zhibi Tuo[Title/Abstract]))) OR ("red yeast rice" [Supplementary Concept] OR "zhibitai" [Supplementary Concept]) |
| **#3** | ("Hydroxymethylglutaryl-CoA Reductase Inhibitors"[Pharmacological Action] OR "Hydroxymethylglutaryl-CoA Reductase Inhibitors"[Mesh] OR "Fluvastatin"[Mesh] OR statin*[Title/Abstract] OR hmg coa reductase[Title/Abstract] OR hydroxymethylglutaryl-coa reductase[Title/Abstract] OR atorvastatin[Title/Abstract] OR lovastatin[Title/Abstract] OR pravastatin[Title/Abstract] OR pitavastatin[Title/Abstract] OR rosuvastatin[Title/Abstract] OR fluvastatin[Title/Abstract] OR simvastatin[Title/Abstract]) |
| **#4** | (((((((controlled clinical trial[Publication Type]) OR (Clinical Trial*[Publication Type])) OR (placebo*[Publication Type])) OR (random*[Publication Type])) OR (allocate*[Publication Type])) OR (assign*[Publication Type])) OR (RCT*[Publication Type])) OR ("Randomized Controlled Trial" [Publication Type]) |
| **#5** | #1 AND #2 AND #3 AND #4 |
| **Search strategies of EMbase (Searched from inception to Nov. 22, 2023 and found 65 literature)** | |
| **#1** | 'dyslipidemia*' OR 'hypercholesterolemia'/exp OR 'familial hyperlipemia'/exp OR 'hyperlipoproteinemia'/exp OR 'hypertriglyceridemia'/exp OR 'dyslipidemia':ti,ab OR 'dyslipoproteinemia*':ti,ab OR 'hyperlipemia*':ti,ab OR 'hyperlipidemia*':ti,ab OR 'lipidemia*':ti,ab OR 'lipemia*':ti,ab OR 'hypercholesterolemia*':ti,ab OR 'high cholesterol level*':ti,ab OR 'cholesterol level*, high':ti,ab OR 'level*, high cholesterol':ti,ab OR 'elevated cholesterol*':ti,ab OR 'cholesterol*, elevated':ti,ab OR 'hypercholesteremia*':ti,ab OR 'hypertriglyceridemia*':ti,ab OR 'combined hyperlipidemia*, familial':ti,ab OR 'familial combined hyperlipidemia*':ti,ab OR 'hyperlipidemia*, familial combined':ti,ab OR 'hyperlipidemia*, multiple lipoprotein-type':ti,ab OR 'lipoprotein-type hyperlipidemia*, multiple':ti,ab OR 'multiple lipoprotein-type hyperlipidemia*':ti,ab OR 'hyperlipoproteinemia*':ti,ab |
| **#2** | 'Monascus'/exp OR 'xuezhikang'/exp OR 'zhibitai'/exp OR 'red yeast rice*':ti,ab OR 'Monascus purpureus':ti,ab OR 'Monascus rubiginosus':ti,ab OR 'Monascus araneosus':ti,ab OR 'Monascus albidus':ti,ab OR 'Monascus anka':ti,ab OR 'Xuezhikang':ti,ab OR 'Xuezhi Kang':ti,ab OR 'Zhibitai':ti,ab OR 'Zhibi Tai':ti,ab OR 'Zhibituo':ti,ab OR 'Zhibi Tuo':ti,ab |
| **#3** | 'hydroxymethylglutaryl coenzyme a reductase inhibitor*'/exp OR 'fluvastatin'/exp OR 'statin (protein)'/exp OR 'hydroxymethylglutaryl coenzyme A reductase*'/exp OR 'atorvastatin':ti,ab OR 'lovastatin':ti,ab OR 'pravastatin':ti,ab OR 'pitavastatin':ti,ab OR 'rosuvastatin':ti,ab OR 'fluvastatin':ti,ab OR 'simvastatin':ti,ab |
| **#4** | 'randomized controlled trial':ti,ab OR 'controlled clinical trial':ti,ab OR 'randomized':ti,ab OR 'placebo':ti,ab OR 'random*':ti,ab OR 'allocate*':ti,ab OR 'assign*':ti,ab OR 'RCT*':ti,ab |
| **#5** | #1 AND #2 AND #3 AND #4 |
| **Search strategies of Web of Science (Searched from inception to Nov. 22, 2023 and found 102 literature)** | |
| **#1** | TS=(Dyslipidemias OR (Hyperlipidemias) OR (Hypercholesterolemia) OR (Hyperlipidemia, Familial Combined) OR (Hyperlipoproteinemias) OR (Hypertriglyceridemia) OR (Dyslipidemia) OR (Dyslipoproteinemia*) OR (Hyperlipemia*) OR (Hyperlipidemia*) OR (Lipidemia*) OR (Lipemia*) OR (Hypercholesterolemia*) OR (High Cholesterol Level*) OR (Cholesterol Level*, High) OR (Level*, High Cholesterol) OR (Elevated Cholesterol*) OR (Cholesterol*, Elevated) OR (Hypercholesteremia*) OR (Hypertriglyceridemia*) OR (Combined Hyperlipidemia*, Familial) OR (Familial Combined Hyperlipidemia*) OR (Hyperlipidemia*, Familial Combined) OR (Hyperlipidemia*, Multiple Lipoprotein-Type) OR (Lipoprotein-Type Hyperlipidemia*, Multiple) OR (Multiple Lipoprotein-Type Hyperlipidemia*) OR (Hyperlipoproteinemia*[Title/Abstract])) |
| **#2** | TS=(Monascus OR (red yeast rice) OR (xuezhikang) OR (zhibitai) OR (Monascus purpureus) OR (Monascus rubiginosus) OR (Monascus araneosus) OR (Monascus albidus) OR (Monascus anka) OR (Xuezhikang) OR (Xuezhi Kang) OR (Zhibitai) OR (Zhibi Tai) OR (Zhibituo) OR (Zhibi Tuo) OR (red yeast rice) OR (zhibitai)) |
| **#3** | TS=(Hydroxymethylglutaryl-CoA Reductase Inhibitors OR (Hydroxymethylglutaryl-CoA Reductase Inhibitors) OR (Fluvastatin) OR (statin*) OR (hmg coa reductase) OR ( hydroxymethylglutaryl-coa reductase) OR (atorvastatin) OR (lovastatin) OR (pravastatin) OR (pitavastatin) OR (rosuvastatin) OR (fluvastatin) OR (simvastatin)) |
| **#4** | TS=(randomized controlled trial OR (controlled clinical trial) OR (randomized) OR (placebo) OR (random*) OR (allocate*) OR (MI) OR (assign*) OR (RCT*)) |
| **#5** | #1 AND #2 AND #3 AND #4 |
| **Search strategies of Cochrane Central Register of Controlled Trials (CENTRAL) (Searched from inception to Nov. 22, 2023 and found 85 literature)** | |
| **#1** | MeSH descriptor: [Dyslipidemias] explode all trees  MeSH descriptor: [Hyperlipidemias] explode all trees  MeSH descriptor: [Hypercholesterolemia] explode all trees  MeSH descriptor: [Hyperlipidemia, Familial Combined] explode all trees  MeSH descriptor: [Hyperlipoproteinemias] explode all trees  MeSH descriptor: [Hypertriglyceridemia] explode all trees  (Dyslipidemia*):ti,ab,kw OR (Dyslipoproteinemia):ti,ab,kw OR (Hyperlipemia*):ti,ab,kw OR (Hyperlipidemia*):ti,ab,kw OR (Lipidemia*):ti,ab,kw OR (Lipemia*):ti,ab,kw OR (Hypercholesterolemia*):ti,ab,kw OR (High Cholesterol Level*):ti,ab,kw OR (Cholesterol Level*, High):ti,ab,kw OR (Level*, High Cholesterol):ti,ab,kw OR (Elevated Cholesterol*):ti,ab,kw OR (Cholesterol*, Elevated):ti,ab,kw OR (Hypercholesteremia*):ti,ab,kw OR (Hypertriglyceridemia*):ti,ab,kw OR (Combined Hyperlipidemia*, Familial):ti,ab,kw OR (Hyperlipidemia*, Familial Combined):ti,ab,kw OR (Familial Combined Hyperlipidemia*):ti,ab,kw OR (Hyperlipidemia*, Multiple Lipoprotein-Type):ti,ab,kw OR (Lipoprotein-Type Hyperlipidemia*, Multiple):ti,ab,kw OR (Multiple Lipoprotein-Type Hyperlipidemia*):ti,ab,kw OR (Hyperlipoproteinemia*):ti,ab,kw |
| **#2** | MeSH descriptor: [Monascus] explode all trees  (red yeast rice*):ti,ab,kw OR (xuezhikang*):ti,ab,kw OR (zhibitai):ti,ab,kw OR (Monascus purpureus):ti,ab,kw OR (Monascus rubiginosus):ti,ab,kw OR (Monascus araneosus):ti,ab,kw OR (Monascus albidus):ti,ab,kw OR (Monascus anka):ti,ab,kw OR (Xuezhikang):ti,ab,kw OR (Xuezhi Kang):ti,ab,kw OR (Zhibitai):ti,ab,kw OR (Zhibi Tai):ti,ab,kw OR (Zhibituo):ti,ab,kw OR (Zhibi Tuo):ti,ab,kw |
| **#3** | MeSH descriptor: [Hydroxymethylglutaryl-CoA Reductase Inhibitors] explode all trees  MeSH descriptor: [Fluvastatin] explode all trees  (hmg coa reductase):ti,ab,kw OR (hydroxymethylglutaryl-coa reductase):ti,ab,kw OR (atorvastatin):ti,ab,kw OR (lovastatin):ti,ab,kw OR (pravastatin):ti,ab,kw OR (pitavastatin):ti,ab,kw OR (rosuvastatin):ti,ab,kw OR (fluvastatin):ti,ab,kw OR (simvastatin):ti,ab,kw |
| **#4** | #1 AND #2 AND #3 |

**Table S4 The criteria of Outcome indicators.**

| **Outcome indicators** | **Clinical efficacy evaluation criteria** |
| --- | --- |
| **Clinical efficacy** | **Obvious effect:** TC decreased by 20% or more, TG decreased by 40% or more, HDL-C increased by 0.26 mmol/L or more, meeting one of the above conditions is obvious effect;  **Effective:** TC decreased by 10%~20%, TG decreased by 20%~40%, HDL-C increased by >0.11~0.26 mmol/L, if one of the above conditions is met, it is effective.  **Invalid:** Those who do not meet the valid criteria are invalid; TC increase ≥10%; If TG increases by more than 10% and HDL-C decreases by 0.1 mmol/L, one of the above conditions is invalid.  **Total effective rate is the significant efficiency plus total effective rate.** |

**Table S5 The reasons of excluded 23studies.**

| **Excluded studies** | **Reasons** |
| --- | --- |
| (Sun, 2021) | Not the study design |
| (Chou et al., 2019) | Not the study design |
| (Chen et al., 2022) | Not the target intervention |
| (Liu and Dong, 2008) | Not the target intervention |
| (Qi et al., 2023) | Not the target intervention |
| (Li et al., 2022) | Not the target intervention |
| (Liu, 2022) | Not the target intervention |
| (Zhang et al., 2021) | Not the target intervention |
| (Dong and Jia, 2008) | Not the target intervention |
| (Li, 2017) | Not the target intervention |
| (Li, 2022) | Not the target intervention |
| (Liu and Yang, 2018) | Not the target intervention |
| (Li and Li, 2020) | Not the target patients |
| (Xue et al., 2019) | Not the target patients |
| (Bai and Cui, 2018) | Not the target patients |
| (Xue et al., 2017) | Not the target patients |
| (Xiao, 2019) | Not the target patients |
| (Liu et al., 2022) | Not the target patients |
| (Li et al., 2022) | Not the target patients |
| (Chen, 2015) | Not the target patients |
| (Jing et al., 2013) | Not the target patients |
| (Zeng and WU, 2019) | Not the target patients |

**References:**

Xue, Y., Tao, L., Wu, S., Wang, G., Qian, L., Li, J., Liao, L., Tang, J., Ji, K., 2017, Red yeast rice induces less muscle fatigue symptom than simvastatin in dyslipidemic patients: a single center randomized pilot trial. BMC Cardiovasc Disord 17, 127.

Bai, M., Cui, D., 2018, Clinical effect of Zhibitai capsule on hyperlipidemia and its influence on blood lipids and hs-CRP. Chinese Journal of Integrative Medicine on Cardio/Cerebrovascular Disease 16, 1250-1252.

Zeng, L., WU, X., 2019, Clinical Effect of Xuezhikang Capsule, Amlodipine and Atorvastatin Calcium in the Treatment of Hypertension and Coronary Heart

Disease. Practical Clinical Journal of Integrated Traditional Chinese and Western Medicine 19, 6-8.

Chen, D., 2015, Efficacy of Xuezhikang Capsule and Atorvastatin on Blood Lipids in Patients with Unstable Angina Pectoris. Chinese Journal of Integrative Medicine on Cardio/Cerebrovascular Disease 13, 914-916.

Chen, X.X., Ma, L., Guo, F., Hao, L., 2022, Influence of Zhebitai Combined with Atorvastatin on hsCRP Level and IMT in Patients with Coronary Heart Disease Complicated with Hyperlipidemia. Inner Mongolia Medical Journal 54, 1305-1308.

Chou, X., Wang, F., Li, F., Liu, W., Ye, Z., 2019, Efect of Xuezhikang Ca psule Combined with Amlodipine Atorvastatin Calcium Tablets on Hypertension Complicated with Coronary

Heart Disease and inflammatorv Factors. Northern pharmacy 16, 18-20.

Dong, M., Jia, W., 2008, Clinical observation of the influence of Xuezhikang on hypersensitive C-reactive protein in patients with cerebral apoplexy and hyperlipidemia. HEILONGJIANG JOURNAL OF TRADITIONAL CHINESE MEDICINE 37, 10-11.

Jing, P., Bai, M., Li, Q., 2013, Effects of atorvastatin combined with zhibhi on lipid and inflammation factor in patients with coronary atherosclerotic heart diease. Journal of Hainan Medical University 19, 1034-1037.

Li, C., 2017, Effectiveness analysis of atorvastatin combined with Xuezhikang in the treatment of hypertension complicated with coronary heart disease. Modern Diagnosis & Treatment 28, 2395-2396.

Li, J., Zhou, X., Wang, Z., Wang, Y., 2022, Clinical effect of Zibitai combined with low-dose Atorvastatin Calcium in the treatment of elderly patients with hyperlipidemia. China Medical Herald 19, 161-164.

Li, Q., Zhu, Y., Wang, K., 2022, Clinical study on Xuezhikang Capsules combined with pravastatin in treatment of chronic heart failure. Drugs & Clinic 37, 2276-2280.

Li, P., Li, Y., 2020, Clinical Efficacy of Zhibitai Capsule in Treating Patients of Dyslipidemia with Phlegm-stasis Binding Pattern. Chinese Journal of Experimental Traditional Medical Formulae 26, 137-141.

Li, X., 2022, Effects of Xuezhikang combined with atorvastatin on the blood pressure, blood lipid and inflammatory factors in elderly patients with hypertension complicated by coronary heart disease. Hebei Medical Journal 44, 2667-2669.

Liu, Q., Zhang, T., Wang, Z., Song, S., Tian, X., 2022, Effect of Zhibitang capsule combined with low-dose atorvastatin on blood lipid level and proportion of regulatory T cells in peripheral blood of patients with unstable angina pectoris. Chinese Journal of Integrative Medicine on Cardio/Cerebrovascular Disease 20, 1284-1287.

Liu, Y., Dong, W., 2008, Effect of Xuezhikang and Simvastatin on hyperlipemia of non-insulin dependent diabete. HAINAN MEDICAL JOURNAL 19, 3-4.

Liu, Y., 2022, The Lipid-lowering Effect of Xuezhikang Combined with Atorvastatin in the Treatment of Coronary Atherosclerotic Heart Disease Complicated with Hyperlipidemia and Its Impact on Cardiovascular Events. China Journal of Pharmaceutical Economics 17, 89-92.

Liu, Y., Yang, X., 2018, The Effects of ZhiBiTai Capsules and Atorvastatin Calcium on Blood Lipid of CHD Patients. Western Journal of Traditional Chinese Medicine 31, 89-91.

Qi, M., Song, Z., Bai, Y., 2023, Clinical trial of Xuezhikang capsule combined with atorvastatin calcium tablet in the treatment of patients with carotid plaque. The Chinese Journal of Clinical Pharmacology 39, 1381-1384.

Sun, Y., 2021, Effect of Xuezhikang combined with atorvastatin on enhanced lipid-lowering and vascular endothelial protection in unstable angina pectoris. Journal Of China Prescription Drug 19, 120-121.

Xiao, J., 2019, Comparison of Clinical Efficacy of Xuezhikang Capsule and Atto Vastatin Calcium Tablets in the Treatment of Senile Lacunar Cerebral Infarction with Hyperlipidemia and Prehypertension. Drug Evaluation 16, 41-43.

Xue, Y., Wang, Z., Yu, Y., Shen, J., Wang, T., Ji, Z., Qin, Z., Zhang, J., 2019, Effect of Chinese and Western medicine combined with lipid regulation on patients with renal artery stenosis hypertension. Chinese Journal of Gerontology 39, 5944-5947.

Zhang, H., Lu, X., Yao, X., 2021, Efects of Atorvastatin Combined with uezhikang Capsules on Vascular Endothelial Function and Levels of L-6TNF-a in Patients with

Hvpertension and Coronary Heart Disease. Practical Clinical Journal of Integrated Traditional Chinese and Western Medicine 21, 64-65.

**Table S6 Trim-and-fill test of ZBTAI combination therapy in reducing TC.**

| **Outcome** | **Effect-size** | **Effect model** | **Before trim-and-fill** | | **After trim-and-fill** | | **Increased research** |
| --- | --- | --- | --- | --- | --- | --- | --- |
|  |  |  | **Pooled estimate** | **95％CI** | **Pooled estimate** | **95％CI** |  |
| ZBTAI plus statin *vs.* statin | MD | FE | -0.300 | -0.357 to -0.242 | 0.741 | 0.700 to 0.785 | 3 |
|  |  | RE | -0.533 | -0.810 to -0.297 | 0.575 | 0.445 to 0.743 |  |

Because of the potential publication bias of ZBTAI combination therapy in reducing TC, we conducted trim-and-fill test analysis to assess the effect of publication bias on the interpretation of the results. The result indicated that several RCTs showing negative findings remained unpublished, which could affect the conclusion.

**Table S7 Meta-regression of ZBTAI combination therapy in reducing LDL-C.**

| **Characteristic** | **Regression coefficient** | **Standard error** | **t** | **P＞\|t\|** | **95% CI** |
| --- | --- | --- | --- | --- | --- |
| Year of publication | -0.008 | 0.014 | -0.57 | 0.577 | -0.038 to 0.022 |
| Sample size | -0.001 | 0.0011 | -1.38 | 0.191 | -0.004 to 0.001 |
| Mean age | -0.0017 | 0.002 | -0.88 | 0.399 | -0.006 to 0.003 |

**Table S8 Trim-and-fill test of ZBTAI combination therapy in reducing LDL-C.**

| **Outcome** | **Effect-size** | **Effect model** | **Before trim-and-fill** | | **After trim-and-fill** | | **Increased research** |
| --- | --- | --- | --- | --- | --- | --- | --- |
|  |  |  | **Pooled estimate** | **95％CI** | **Pooled estimate** | **95％CI** |  |
| ZBTAI plus statin *vs.* statin | MD | FE | -0.325 | -0.369 to -0.28 | 0.723 | 0.691to 0.755 | 3 |
|  |  | RE | -0.467 | -0.619 to -0.32 | 0.627 | 0.538 to 729 |  |

Because of the potential publication bias of ZBTAI combination therapy in reducing LDL-C, we conducted trim-and-fill test analysis to assess the effect of publication bias on the interpretation of the results. The result indicated that several RCTs showing negative findings remained unpublished, which could affect the conclusion.

**Table S9 Meta-regression of ZBTAI combination therapy in reducing TG.**

| **Characteristic** | **Regression coefficient** | **Standard error** | **t** | **P＞\|t\|** | **95% CI** |
| --- | --- | --- | --- | --- | --- |
| Year of publication | -0.008 | 0.126 | -0.62 | 0.545 | –0.035 to 0.019 |
| Sample size | -0.0005 | 0.0098 | -0.55 | 0.595 | –0.003 to 0.0016 |
| Mean age | -0.0008 | 0.0019 | -0.44 | 0.667 | –0.005 to 0.003 |

**Table S10 Meta-regression of red yeast rice-containing CCPPs in reducing HDL-C.**

| **Characteristic** | **Regression coefficient** | **Standard error** | **t** | **P＞\|t\|** | **95% CI** |
| --- | --- | --- | --- | --- | --- |
| **XZK combination therapy** | | | | | |
| Year of publication | 0.004 | 0.004 | 1.01 | 0.337 | -0.004 to 0.0115 |
| Sample size | -0.00002 | 0.0001 | -0.14 | 0.892 | -0.0003 to 0.002 |
| Mean age | -0.002 | 0.003 | -0.54 | 0.6 | -0.009 to 0.005 |
| **ZBTUO combination therapy** | | | | | |
| Year of publication | 0.002 | 0.006 | 0.29 | 0.798 | -0.025 to 0.029 |
| Sample size | 0.00003 | 0.001 | 0.03 | 0.979 | -0.004 to 0.004 |
| Mean age | -0.0001 | 0.003 | -0.03 | 0.979 | -0.014 to 0.014 |
| **ZBTAI combination therapy** | | | | | |
| Year of publication | -0.0007 | 0.006 | -0.12 | 0.908 | -0.014 to 0.0013 |
| Sample size | -0.0004 | 0.0003 | -1.21 | 0.246 | -0.001 to 0.0003 |
| Mean age | 0.0001 | 0.0008 | 0.12 | 0.909 | -0.002 to 0.002 |

**Table S11 Trim-and-fill test of XZK combination therapy in reducing HDL-C.**

| **Outcome** | **Effect-size** | **Effect model** | **Before trim-and-fill** | | **After trim-and-fill** | | **Increased research** |
| --- | --- | --- | --- | --- | --- | --- | --- |
|  |  |  | **Pooled estimate** | **95％CI** | **Pooled estimate** | **95％CI** |  |
| XZKplus statin *vs.* statin | MD | FE | 0.195 | 0.171 to 0.219 | 1.173 | 1.147 to 1.199 | 5 |
|  |  | RE | 0.234 | 0.176 to0.292 | 1.179 | 1.108 to 1.255 |  |

Because of the potential publication bias of XZK combination therapy in reducing HDL-C, we conducted trim-and-fill test analysis to assess the effect of publication bias on the interpretation of the results. The result indicated that this publication bias did not affect the estimates, although several RCTs showing negative findings remained unpublished.

**Table S12 Details of the adverse event occurred in the included studies.**

| **Study** | **T** | **C** |
| --- | --- | --- |
| Zou et al. (2017) | no | skeletal muscle pain (10), joint swelling (15),  muscle fatigue (9) |
| Shi et al. (2018) | gastrointestinal reaction (2) | gastrointestinal reaction (5) |
| Yu et al. (2021) | joint swelling (1) | skeletal muscle pain (2), joint swelling (3),  muscle fatigue (1) |
| Wang et al. (2012) | gastrointestinal reaction (1) | gastrointestinal reaction (8) |
| Liu et al. (2018) | no | joint swelling (1) |
| Zhang et al. (2010) | no | gastrointestinal reaction (20),  slightly increased of ALT (8), muscle pain (1) |
| Fu et al. (2017) | gastrointestinal reaction (3), flushed face (2) | gastrointestinal reaction (3),  abnormal kidney function (1) |
| Ma et al. (2019) | no | no |
| Su et al. (2021) | gastrointestinal reaction (2), flushed face (2) | gastrointestinal reaction (3) |
| Li et al. (2023) | muscle pain (1),  abnormal liver function (1), dizziness (1) | muscle pain (2),  abnormal liver function (2), gastrointestinal reaction (1) |
| Yuan et al. (2023) | gastrointestinal reaction (2), | rashes (1), gastrointestinal reaction (1), slightly increased of ALT (1) |
| Chen et al. (2022) | no | no |
| Tan et al. (2021) | palpitations (2),  flushed face (1) | palpitations (1),  flushed face (4) |
| Xiong et al. (2019) | no | no |
| Tan et al. (2020) | dizziness and headache(1), muscle fatigue (2), gastrointestinal reaction (1) | dizziness and headache(5), muscle fatigue (4), gastrointestinal reaction (2), rashes (1) |
| He et al. (2020) | gastrointestinal reaction (3) | gastrointestinal reaction (5) |
| Ma et al. (2018) | gastrointestinal reaction (1), abnormal liver function (1) | gastrointestinal reaction (3), abnormal liver function (2) |
| Wang et al. (2015) | abnormal liver function (2) | abnormal liver function (2) |

**Table S13 Summary of findings.**

| NO. | Study design | Certainty assessment | | | | | Summary of results | | | | | | Importance |
| --- | --- | --- | --- | --- | --- | --- | --- | --- | --- | --- | --- | --- | --- |
|  |  | Risk of bias | Inconsistency | Indirectness | Imprecision | Others | No of patients | | Effect (95%CI) | | Certainty | |  |
|  |  |  |  |  |  |  | T | C | Relative | Absolute |  |  |  |
| **XZK combination therapy in reducing TC** | | | | | | | | | | | | | |
| 14 | RCT | not Serious | Serious **^b^** | not Serious | not Serious | not Serious | 1049 | 1045 | - | MD = -0.63  (-0.83 to -0.44) | ⨁⨁⨁◯ | [moderate](http://www.baidu.com/link?url=DIGeccXOKFG4xoRsRpF0Gbl9Y-yGELRUVBKY-Z5K8c6klZK-kzKIKGFxGJpGzVp_iHlWiUBDYE9l2hOtP9HRlJSOgcdaUKMRUJQRxocaxxu) | Critical |
| **ZBTAI combination therapy in reducing TC** | | | | | | | | | | | | | |
| 15 | RCT | not Serious | Serious **^b^** | not Serious | not Serious | Serious **^d^** | 738 | 744 | - | MD = -0.55  (-0.81 to -0.30) | ⨁⨁◯◯ | Low | Critical |
| **ZBTUO combination therapy in reducing TC** | | | | | | | | | | | | | |
| 4 | RCT | not Serious | not Serious | not Serious | not Serious | Serious **^e^** | 261 | 261 | - | MD = -0.65  (-0.82 to -0.48) | ⨁⨁⨁◯ | [moderate](http://www.baidu.com/link?url=DIGeccXOKFG4xoRsRpF0Gbl9Y-yGELRUVBKY-Z5K8c6klZK-kzKIKGFxGJpGzVp_iHlWiUBDYE9l2hOtP9HRlJSOgcdaUKMRUJQRxocaxxu) | Critical |
| **XZK combination therapy in reducing TG** | | | | | | | | | | | | | |
| 12 | RCT | not Serious | Serious **^b^** | not Serious | not Serious | not Serious | 958 | 954 | - | MD = -0.31  (-0.41 to -0.21) | ⨁⨁◯◯ | Low | Critical |
| **ZBTAI combination therapy in reducing TG** | | | | | | | | | | | | | |
| 15 | RCT | not Serious | Serious **^b^** | not Serious | not Serious | not Serious | 738 | 744 | - | MD = -0.35  (-0.45 to -0.24) | ⨁⨁⨁◯ | [moderate](http://www.baidu.com/link?url=DIGeccXOKFG4xoRsRpF0Gbl9Y-yGELRUVBKY-Z5K8c6klZK-kzKIKGFxGJpGzVp_iHlWiUBDYE9l2hOtP9HRlJSOgcdaUKMRUJQRxocaxxu) | Critical |
| **ZBTUO combination therapy in reducing TG** | | | | | | | | | | | | | |
| 4 | RCT | not Serious | not Serious | not Serious | not Serious | Serious **^e^** | 261 | 261 | - | MD = -0.28  (-0.39 to -0.17) | ⨁⨁⨁◯ | [moderate](http://www.baidu.com/link?url=DIGeccXOKFG4xoRsRpF0Gbl9Y-yGELRUVBKY-Z5K8c6klZK-kzKIKGFxGJpGzVp_iHlWiUBDYE9l2hOtP9HRlJSOgcdaUKMRUJQRxocaxxu) | Critical |
| **XZK combination therapy in reducing LDL-C** | | | | | | | | | | | | | |
| 14 | RCT | not Serious | Serious **^b^** | not Serious | not Serious | not Serious | 1049 | 1045 | - | MD = -0.37  (-0.52 to -0.22) | ⨁⨁⨁◯ | [moderate](http://www.baidu.com/link?url=DIGeccXOKFG4xoRsRpF0Gbl9Y-yGELRUVBKY-Z5K8c6klZK-kzKIKGFxGJpGzVp_iHlWiUBDYE9l2hOtP9HRlJSOgcdaUKMRUJQRxocaxxu) | Critical |
| **ZBTAI combination therapy in reducing LDL-C** | | | | | | | | | | | | | |
| 15 | RCT | not Serious | Serious **^b^** | not Serious | not Serious | Serious **^d^** | 738 | 744 | - | MD = -0.47  (-0.62 to -0.32) | ⨁⨁◯◯ | Low | Critical |
| **ZBTUO combination therapy in reducing LDL-C** | | | | | | | | | | | | | |
| 4 | RCT | not Serious | not Serious | not Serious | not Serious | Serious **^e^** | 261 | 261 | - | MD = -0.51  (-0.64 to -0.37) | ⨁⨁⨁◯ | [moderate](http://www.baidu.com/link?url=DIGeccXOKFG4xoRsRpF0Gbl9Y-yGELRUVBKY-Z5K8c6klZK-kzKIKGFxGJpGzVp_iHlWiUBDYE9l2hOtP9HRlJSOgcdaUKMRUJQRxocaxxu) | Critical |
| **XZK combination therapy in reducing HDL-C** | | | | | | | | | | | | | |
| 12 | RCT | not Serious | Serious **^b^** | not Serious | not Serious | Serious **^d^** | 827 | 825 | - | MD = 0.23  (0.18 to 0.29) | ⨁⨁◯◯ | Low | Critical |
| **ZBTAI combination therapy in reducing HDL-C** | | | | | | | | | | | | | |
| 15 | RCT | not Serious | Serious **^b^** | not Serious | not Serious | not Serious | 738 | 744 | - | MD = 0.21  (0.14 to 0.28) | ⨁⨁⨁◯ | [moderate](http://www.baidu.com/link?url=DIGeccXOKFG4xoRsRpF0Gbl9Y-yGELRUVBKY-Z5K8c6klZK-kzKIKGFxGJpGzVp_iHlWiUBDYE9l2hOtP9HRlJSOgcdaUKMRUJQRxocaxxu) | Critical |
| **ZBTUO combination therapy in reducing HDL-C** | | | | | | | | | | | | | |
| 4 | RCT | not Serious | Serious **^b^** | not Serious | not Serious | Serious **^e^** | 261 | 261 | - | MD = 0.23  (0.18 to 0.29) | ⨁⨁◯◯ | Low | Critical |
| **XZK combination therapy in clinical efficacy** | | | | | | | | | | | | | |
| 9 | RCT | not Serious | not Serious | not Serious | not Serious | not Serious | 785 | 676 | RR = 1.16  (1.12 to 1.20) | - | ⨁⨁⨁⨁ | High | Important |
| **ZBTAI combination therapy in clinical efficacy** | | | | | | | | | | | | | |
| 5 | RCT | not Serious | not Serious | not Serious | not Serious | Serious **^e^** | 268 | 275 | RR = 1.17  (1.10 to 1.25) | - | ⨁⨁⨁◯ | [moderate](http://www.baidu.com/link?url=DIGeccXOKFG4xoRsRpF0Gbl9Y-yGELRUVBKY-Z5K8c6klZK-kzKIKGFxGJpGzVp_iHlWiUBDYE9l2hOtP9HRlJSOgcdaUKMRUJQRxocaxxu) | Important |
| **ZBTUO combination therapy in clinical efficacy** | | | | | | | | | | | | | |
| 4 | RCT | not Serious | not Serious | not Serious | not Serious | Serious **^e^** | 261 | 261 | RR = 1.15  (1.08 to 1.23) | - | ⨁⨁⨁◯ | [moderate](http://www.baidu.com/link?url=DIGeccXOKFG4xoRsRpF0Gbl9Y-yGELRUVBKY-Z5K8c6klZK-kzKIKGFxGJpGzVp_iHlWiUBDYE9l2hOtP9HRlJSOgcdaUKMRUJQRxocaxxu) | Important |

Abbreviations: RCT, randomized controlled trial; MD, mean difference; RR, relative risk; CI, confidence interval.

a. Downgrade by one level: More than 25% of the studies were those with a high risk of overall bias.

b. Downgrade by one level: Heterogeneity among the studies was fairly high.

c. Downgrade by one level: The optimal information sample size was less than 400 participants.

d. Downgrade by one level: There was a risk of publication bias.

e. Downgrade by one level: The number of RCTs was s less than 6.

**Table S14 Methodological quality assessment**

| **Quality evaluation** | **Y/N** | **Location where item is reported** |
| --- | --- | --- |
| 1. Did the research questions and inclusion criteria for the review include the components of PICO? | Y |  |
| 1. Did the report of the review contain an explicit statement that the review methods were established prior to the conduct of the review and did the report justify any significant deviations from the protocol? | Y | without deviations |
| 1. Did the review authors explain their selection of the study designs for inclusion in the review? | Y |  |
| 1. Did the review authors use a comprehensive literature search strategy? | Y | Supplement Table S3 |
| 1. Did the review authors perform study selection in duplicate? | Y |  |
| 1. Did the review authors perform data extraction in duplicate? | Y |  |
| 1. Did the review authors provide a list of excluded studies and justify the exclusions? | Y | Supplementary Table S5 |
| 1. Did the review authors describe the included studies in adequate detail? | Y |  |
| 1. Did the review authors use a satisfactory technique for assessing the risk of bias (RoB) in individual studies that were included in the review? | Y |  |
| 1. Did the review authors report on the sources of funding for the studies included in the review? | Y |  |
| 1. If meta-analysis was performed, did the review authors use appropriate methods for statistical combination of results? | Y |  |
| 1. If meta-analysis was performed, did the review authors assess the potential impact of RoB in individual studies on the results of the meta-analysis or other evidence synthesis? | Y |  |
| 1. Did the review authors account for RoB in primary studies when interpreting/discussing the results of the review? | Y |  |
| 1. Did the review authors provide a satisfactory explanation for, and discussion of, any heterogeneity observed in the results of the review? | Y |  |
| 1. If they performed quantitative synthesis did the review authors carry out an adequate investigation of publication bias (small study bias) and discuss its likely impact on the results of the review? | Y |  |
| 1. Did the review authors report any potential sources of conflict of interest, including any funding they received for conducting the review? | Y | Supplementary |

**Table S15 the risk of bias in this systematic review.**

| **Phase 1: Assessing relevance** | | | Y |  |
| --- | --- | --- | --- | --- |
| **Phase 2. Identifying concerns about bias in the review process** | **Domain 1. Study eligibility criteria** | Q1. Did the review adhere to predefined objectives and eligibility criteria? | Y |  |
|  |  | Q2. Were the eligibility criteria appropriate for the review question? | Y |  |
|  |  | Q3.Were eligibility criteria unambiguous? | Y |  |
|  |  | Q4. Were all restrictions in eligibility criteria based on study characteristics appropriate? | Y |  |
|  |  | Q5. Were any restrictions in eligibility criteria based on sources of information appropriate? | Y |  |
|  |  | **Risk rating** | Low |  |
|  | **Domain 2. Identification and**  **selection of studies** | Q1. Did the search include an appropriate range of databases/electronic sources for published and unpublished reports? | Y |  |
|  |  | Q2. Were methods additional to database searching used to identify relevant reports? | Y |  |
|  |  | Q3. Were the terms and structure of the search strategy likely to retrieve as many eligible studies as possible? | Y | Supplement Table S3 |
|  |  | Q4. Were restrictions based on date.publication format, or language appropriate? | Y |  |
|  |  | Q5. Were efforts made to minimize error in selection of studies? | Y |  |
|  |  | **Risk rating** | Low |  |
|  | **Domain 3. Data collection and study appraisal** | Q1. Were efforts made to minimize error in data collection? | Y |  |
|  |  | Q2. Were sufficient study characteristics available for both review authors and readers to be able to interpret the results? | Y |  |
|  |  | Q3. Were all relevant study results collected for use in the synthesis? | Y |  |
|  |  | Q4. Was risk of bias (or methodological quality) formally assessed using appropriate criteria? | Y |  |
|  |  | Q5. Were efforts made to minimize error in risk of bias assessment? | Y |  |
|  |  | **Risk rating** | Low |  |
|  | **Domain 4. Synthesis and findings** | Q1. Did the synthesis include all studies that it should? | Y |  |
|  |  | Q2. Were all predefined analyses reported or departures explained? | Y |  |
|  |  | Q3. Was the synthesis appropriate given the nature and similarity in the research questions, study designs, and outcomes across included studies? | Y |  |
|  |  | Q4. Was between-study variation minimal or addressed in the synthesis? | Y |  |
|  |  | Q5. Were the findings robust, for example.as demonstrated through funnel plot or sensitivity analyses? | Y |  |
|  |  | Q6. Were biases in primary studies minimal or addressed in the synthesis? | Y |  |
|  |  | **Risk rating** | Low |  |
| **Phase 3. Risk of bias in the review** | | Q1. Did the interpretation of findings address all of the concerns identified in domains 1 to 4? | Y |  |
|  |  | Q2. Was the relevance of identified studies to the review's research question appropriately considered? | Y |  |
|  |  | Q3. Did the reviewers avoid emphasizing results on the basis of their statistical significance? | Y |  |
|  | | **Risk rating** | Low |  |

**Fig. S1 Trim-and-fill test of ZBTAI combination therapy in reducing TC. (To assess the effect of publication bias on the interpretation of the results).**


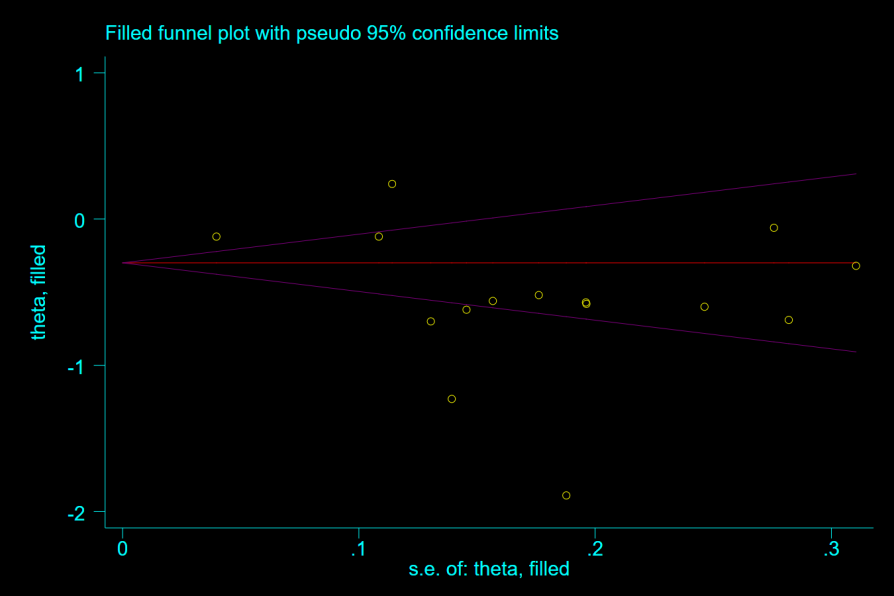


**Fig. S2 Publication bias of red yeast rice-containing CCPPs on LDL-C.**

(A) Egger's test quantified the publication bias of XZK on LDL-C. (B) Forest plot of XZK on LDL-C. (C) Egger's test quantified the publication bias of ZBTAI on LDL-C. (D) Forest plot of ZBTAI on LDL-C.


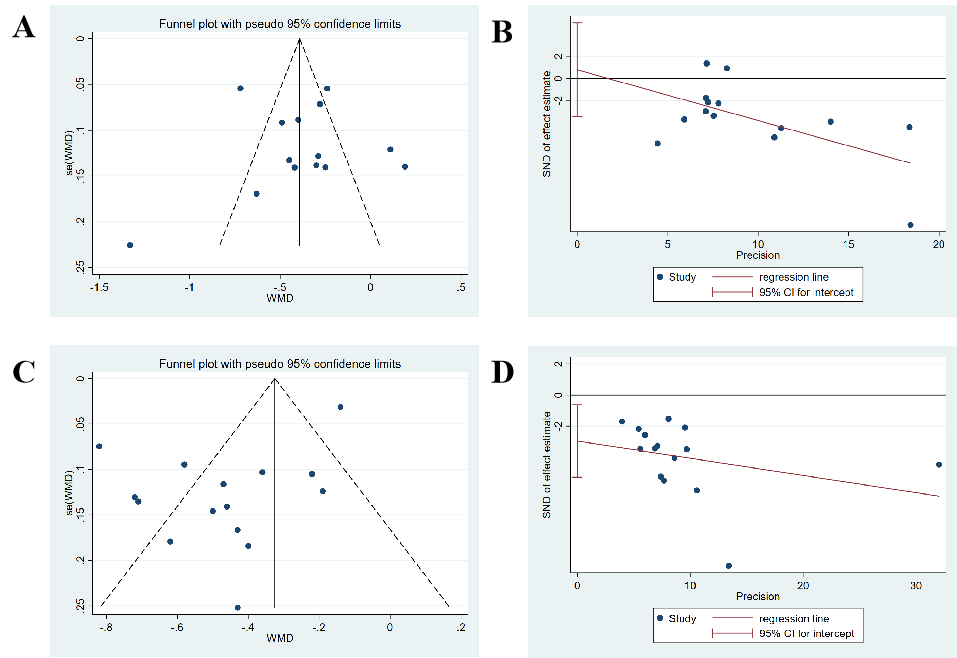


**Fig. S3 Trim-and-fill test of ZBTAI combination therapy in reducing LDL-C.**


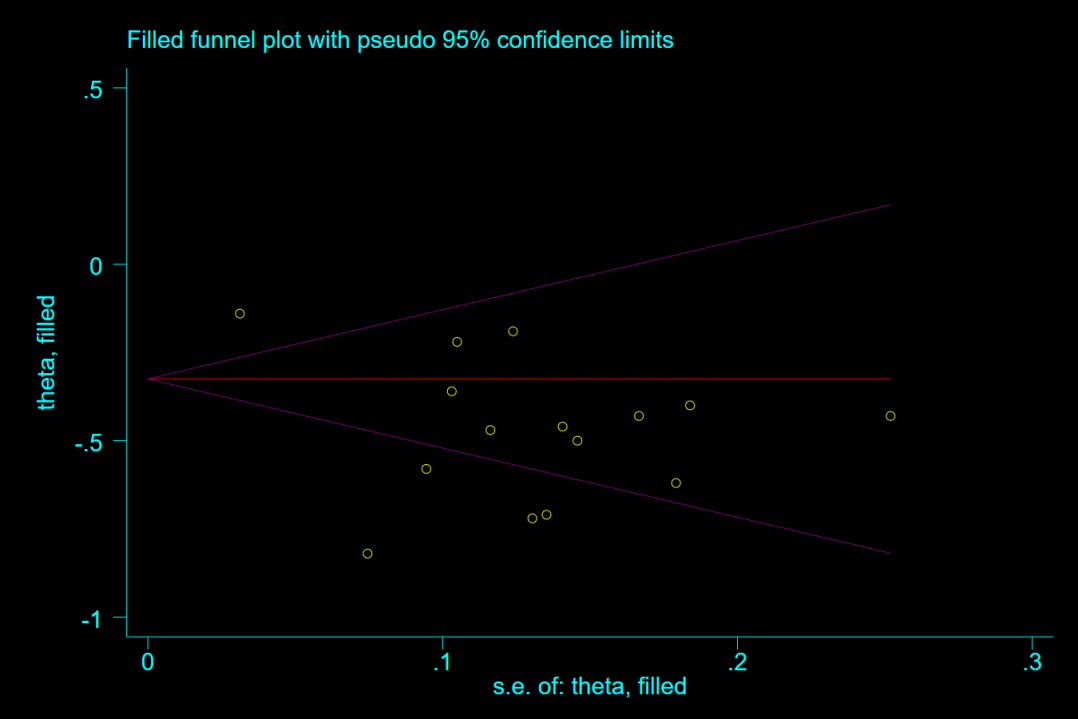


**Fig. S4 Publication bias of red yeast rice-containing CCPPs on TG.**

(A) Forest plot of XZK on TG. (B) Egger's test quantified the publication bias of XZK on TG. (C) Forest plot of ZBTAI on TG. (D) Egger's test quantified the publication bias of ZBTAI on TG.


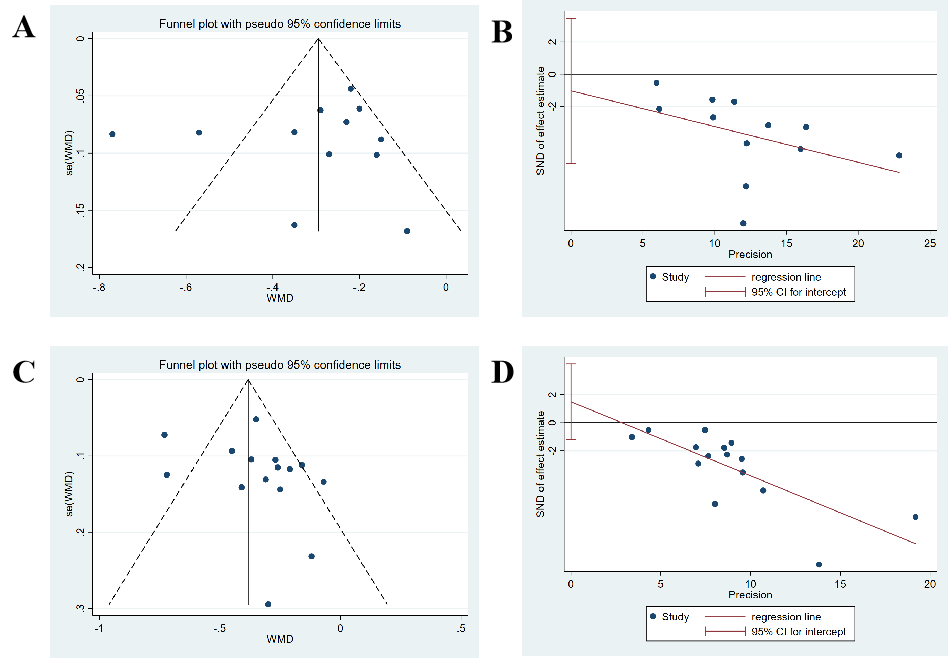


**Fig. S5 Publication bias of red yeast rice-containing CCPPs on HDL-C.**

(A) Forest plot of XZK on HDL-C. (B) Egger's test quantified the publication bias of XZK on HDL-C. (C) Forest plot of ZBTAI on HDL-C. (D) Egger's test quantified the publication bias of ZBTAI on HDL-C.


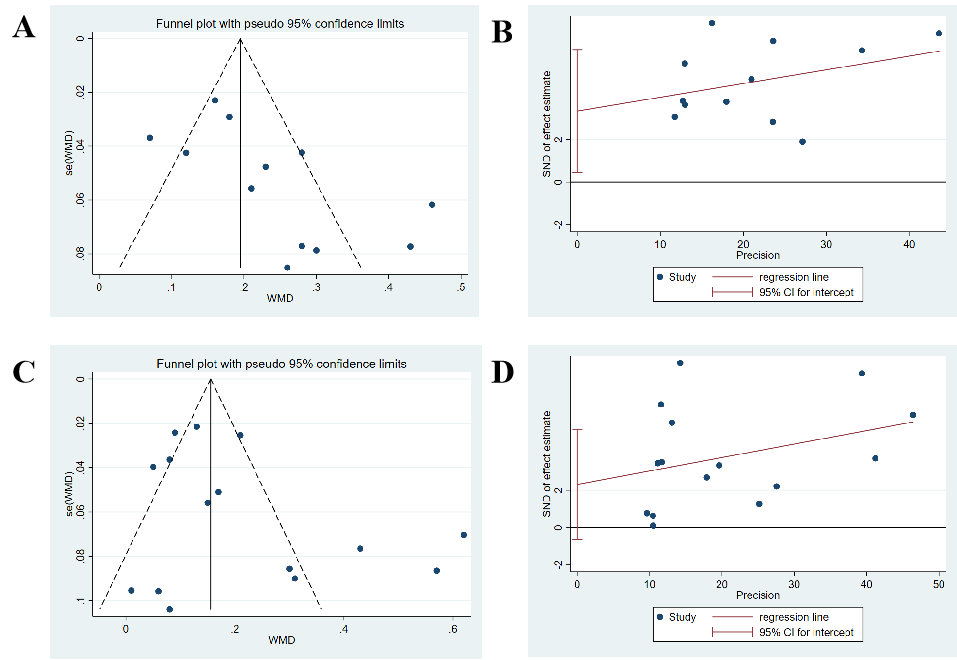


**Fig. S6 Trim-and-fill test of XZK combination therapy in reducing HDL-C.**


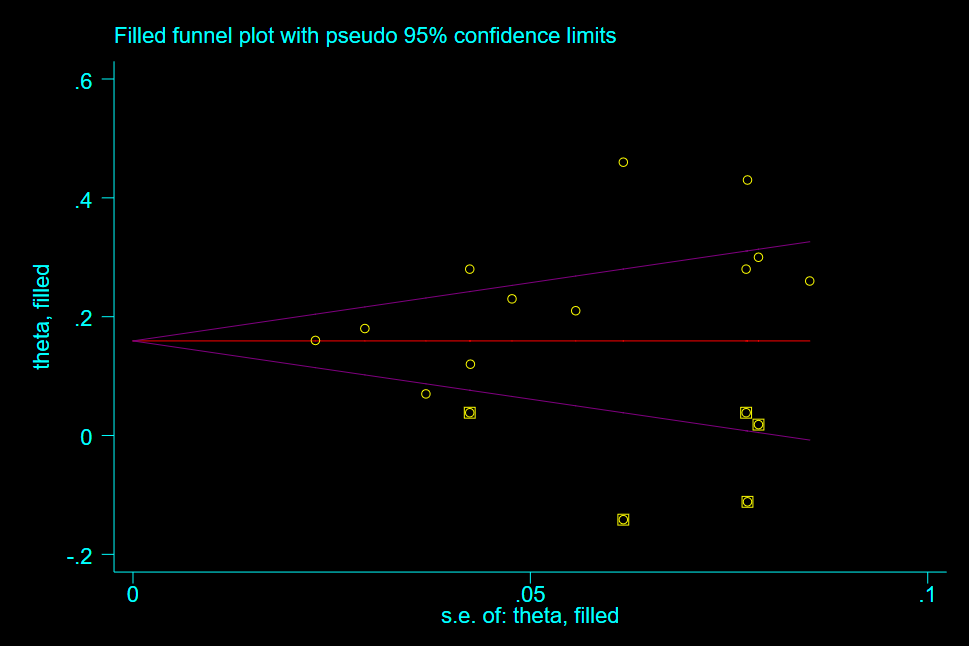

Supplement: Supplementary file 1 [file DataSheet1.doc]
